# Immune and epithelial determinants of age-related risk and alveolar injury in fatal COVID-19

**DOI:** 10.1172/jci.insight.157608

**Published:** 2022-06-08

**Authors:** Michael Chait, Mine M. Yilmaz, Shanila Shakil, Amy W. Ku, Pranay Dogra, Thomas J. Connors, Peter A. Szabo, Joshua I. Gray, Steven B. Wells, Masaru Kubota, Rei Matsumoto, Maya M.L. Poon, Mark E. Snyder, Matthew R. Baldwin, Peter A. Sims, Anjali Saqi, Donna L. Farber, Stuart P. Weisberg

**Affiliations:** 1 Department of Pathology and Cell Biology,; 2 Department of Microbiology and Immunology,; 3 Department of Pediatrics,; 4 Department of Systems Biology, and; 5 Department of Surgery, Columbia University Irving Medical Center, New York, New York, USA.; 6 Medical Scientist Training Program, Columbia University, New York, New York, USA.; 7 Department of Medicine, University of Pittsburgh, Pittsburgh, Pennsylvania, USA.; 8 Department of Medicine and; 9 Department of Biochemistry and Molecular Biophysics, Columbia University Irving Medical Center, New York, New York, USA.

**Keywords:** Aging, COVID-19, Macrophages, Pulmonary surfactants, T cells

## Abstract

Respiratory failure in COVID-19 is characterized by widespread disruption of the lung’s alveolar gas exchange interface. To elucidate determinants of alveolar lung damage, we performed epithelial and immune cell profiling in lungs from 24 COVID-19 autopsies and 43 uninfected organ donors ages 18–92 years. We found marked loss of type 2 alveolar epithelial (T2AE) cells and increased perialveolar lymphocyte cytotoxicity in all fatal COVID-19 cases, even at early stages before typical patterns of acute lung injury are histologically apparent. In lungs from uninfected organ donors, there was also progressive loss of T2AE cells with increasing age, which may increase susceptibility to COVID-19–mediated lung damage in older individuals. In the fatal COVID-19 cases, macrophage infiltration differed according to the histopathological pattern of lung injury. In cases with acute lung injury, we found accumulation of CD4^+^ macrophages that expressed distinctly high levels of T cell activation and costimulation genes and strongly correlated with increased extent of alveolar epithelial cell depletion and CD8^+^ T cell cytotoxicity. Together, our results show that T2AE cell deficiency may underlie age-related COVID-19 risk and initiate alveolar dysfunction shortly after infection, and we define immune cell mediators that may contribute to alveolar injury in distinct pathological stages of fatal COVID-19.

## Graphical abstract

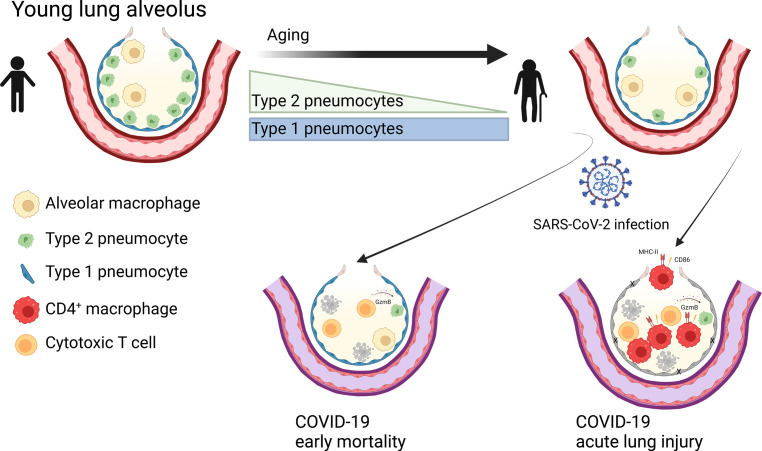

## Introduction

SARS-CoV-2 is unique among viral respiratory pathogens in its capacity to rapidly induce respiratory failure in adults. The risk for death by COVID-19 increases progressively and dramatically with age (1) such that individuals over 65 years of age comprise more than 80% of deaths from COVID-19 (2, 3), while male sex (4), hypertension, and diabetes mellitus (5) are also risk factors for severe COVID-19.

The primary cause of death in patients with severe COVID-19 is lung damage leading to failure of pulmonary gas exchange (6–8). Although SARS-CoV-2 enters the respiratory tract by infecting cells of nasal and large airway epithelium that have the highest expression of the viral entry receptor angiotensin-converting enzyme 2 (9), fatal cases often show severe damage to the distal lung, particularly the small airspaces — alveoli — that comprise the lung’s gas exchange interface (10–13). The mechanisms of alveolar injury and age-related susceptibility to lung damage and mortality from SARS-CoV-2 infection remain poorly defined.

Due to the uniquely rapid course of respiratory failure in COVID-19, deaths often occur precipitously, before supportive care can be established. Patients who receive advanced supportive care, such as mechanical ventilation, often do not recover pulmonary function and die after protracted intervals of hospitalization (4). Early mortality cases — typically hours or days after hospitalization (4) — often have severe hypoxia, precipitous clinical decline, and death without either prominent pulmonary immune infiltrates or typical histological patterns of acute lung injury (ALI) (11, 14–17). These cases likely reflect the earliest stages of viral infection and associated inflammation, and they show higher viral burden and high levels of proinflammatory cytokines and interferon-stimulated genes (ISGs) (11). Late mortality cases — typically 1–2 weeks after hospitalization (4) — show increased pulmonary immune infiltration (14, 18) and typical histological patterns of ALI affecting the alveolar epithelial lining, most commonly diffuse alveolar damage (DAD) (10, 11, 19). Thus, the lung injury pattern and immune infiltration in COVID-19 occurs along a continuum dependent on the time postinfection, with early mortality cases reflecting the earliest stages of lung injury and late mortality cases showing advanced lung damage and dysfunctional repair mechanisms (14).

The alveolar gas exchange interface comprises type 1 alveolar epithelial (T1AE) cells with flattened morphology that actively participate in gas exchange and type 2 alveolar epithelial (T2AE) cells with cuboidal morphology that produce pulmonary surfactant required for maintaining alveolar surface tension. Notably, T2AE cells are less differentiated than T1AE cells and possess progenitor capacity for self-renewal and repair of alveolar epithelial damage (20, 21).

There is substantial evidence that the immunopathological response to SARS-CoV-2 infection plays a major role in alveolar epithelial destruction (12, 18, 22–25). Single-cell RNA-Seq studies of COVID-19 patient lung and airway washings and single-nucleus RNA-Seq studies of COVID-19 autopsy samples reveal severe dysregulation of both epithelial and immune function (12, 22, 23). There is evidence for unregulated tissue inflammatory responses with aberrantly activated monocytes/macrophages and T cells as well as impaired alveolar epithelial cell function and regeneration (12, 23, 26). Defining the interrelationships between alveolar epithelial damage and tissue immune cells at distinct immunopathological stages of COVID-19 can elucidate mechanisms of respiratory failure to better optimize COVID-19 medical management and mitigation efforts. In addition, a better understanding of the factors involved in alveolar injury can help advance our understanding of many other lung diseases that cause respiratory failure by disrupting alveolar epithelium (27).

Here we have performed gene expression studies coupled with epithelial and immune cell profiling focused on the immunopathological processes at the lung’s alveolar gas exchange interface in 24 COVID-19 autopsies and 43 uninfected organ donors. We identify marked and selective T2AE cell loss and increased perialveolar lymphocyte cytotoxicity as defining elements of early lung tissue changes in fatal COVID-19 that are manifest prior to appearance of the typical histological ALI patterns. In the lungs of uninfected individuals, we show that selective depletion of T2AE cells correlates with increasing age, suggesting that lower baseline T2AE cell density may increase risk in older individuals for severe lung damage from COVID-19. In the lungs of fatal COVID-19 cases with ALI, we identify prominent infiltration of CD4^+^ macrophages expressing high levels of T cell activation and costimulation genes, which strongly correlates with increased extent of alveolar epithelial cell depletion and CD8^+^ T cell cytotoxicity. Together, our results provide important insights into the dynamics and interrelationships of alveolar epithelial cells with age and immune cell infiltration and elucidate how immune cells might orchestrate alveolar injury in fatal COVID-19.

## Results

### Histopathology of early and late COVID-19 mortality from an autopsy series.

**Figure 1:**
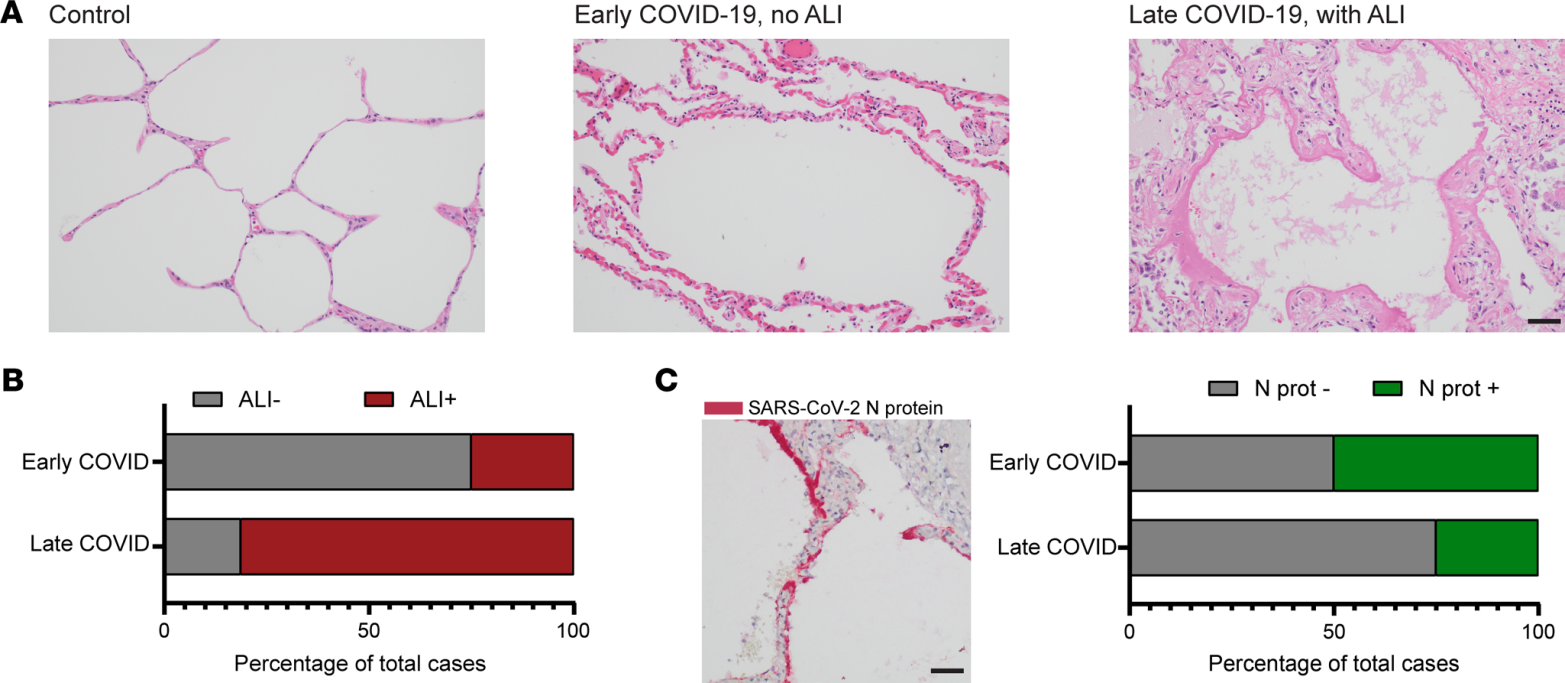
Histopathological findings in early and late COVID-19 mortality. (**A**) H&E-stained sections (20× original magnification fields) demonstrating the predominant histopathological changes seen in uninfected lung (left panel, control), early COVID-19 mortality (middle panel, <10 days symptomatic interval), and late COVID-19 mortality (right panel, >10 days symptomatic interval). (**B**) Bar plots depicting the percentage of early COVID-19 mortality (*n* = 8) and late COVID-19 mortality cases (*n* = 16) showing predominant histological patterns of acute lung injury (ALI, shown in red). (**C**) Representative SARS-CoV-2 nucleocapsid protein (N protein) stain is shown (left) with bar plots depicting the percentage of early (*n* = 8) and late (*n* = 16) COVID-19 mortality cases that were histologically positive for the SARS-CoV-2 N protein (shown in green, right). Black scale bar: 50 μm.

To define histopathologic changes associated with early and late COVID-19 mortality, fatal COVID-19 cases (*n* = 24) were selected from among a previously defined cohort of postmortem examinations performed at NewYork-Presbyterian Hospital on patients who died of COVID-19 between March and June of 2020 (11). All cases were confirmed SARS-CoV-2 positive by PCR with either pre- or postmortem testing and had availability of paraffin-embedded lung tissue blocks without autolysis. The fatal COVID-19 cases largely comprised older adults ranging in age from 57 to 93 (median age, 73), predominantly men (75%), with the majority Hispanic or African American (54%). Comorbidities previously shown to be associated with COVID-19 mortality were present in the vast majority of these cases, including hypertension (96%), diabetes (50%), and heart disease (46%). The median time from symptom onset to death was 16.5 days (range, 1–42 days). Consistent with previous reports showing distinct early and late peaks of COVID-19 mortality (4, 14), 1 in 3 of the patients (8/24) in our cohort presented in rapidly deteriorating clinical condition, with death within 5 days of hospitalization (median, 0.5; range, 0–5 days) and 10 days of symptom onset (median, 2.5; range, 1–9 days). The remaining patients (16/24) displayed more gradual clinical decline, with death occurring after a more prolonged symptomatic period (median, 19.5; range, 10–43 days) (Supplemental Table 1; supplemental material available online with this article; https://doi.org/10.1172/jci.insight.157608DS1). Otherwise, the profile of clinical characteristics and comorbidities was similar between the early and late mortality groups, with all patients showing evidence of hypoxemia based on oxygen saturation or partial pressure of oxygen (Supplemental Table 1). Histopathological assessment of the postmortem examinations (Figure 1A) revealed patterns of ALI (see Methods) (11, 28), predominately DAD in 81.25% of the late COVID-19 mortality cases (13/16 cases) and 25% in the early COVID-19 mortality cases (2/8 cases) (Figure 1B). In 75% of the early mortality cases, signs of vascular congestion and capillary proliferation were observed (Supplemental Table 1 and Figure 1A) consistent with previous reports (11, 13). Staining for SARS-CoV-2 nucleocapsid protein (N protein) was positive in 50% (4/8) of the early COVID-19 mortality cases and 25% (4/16) of the late COVID-19 mortality cases (Figure 1C). In N protein–positive areas (Figure 1C), staining localized mostly to hyaline membranes and apical surfaces of pneumocytes lining the alveolar septa, consistent with previous reports (14, 29). Simple logistic regression analysis showed a significantly increased probability of ALI (odds ratio = 1.124, CI = 1.022–1.1277, *P* = 0.0386) and decreased probability of SARS-CoV-2 N protein positivity (odds ratio = 0.9096, CI = 0.8079–0.9955, *P* = 0.0128) associated with increased symptomatic interval. These results show that widespread ALI is most frequently associated with late COVID-19 mortality and is often not present in early COVID-19 mortality.

### ISGs and loss of surfactant transcripts in early COVID-19 mortality.

To define changes in gene expression associated with early and late COVID-19 mortality in bulk lung tissue, we used RNA extracted from the FFPE archival lung tissue to profile pulmonary gene expression across 8 late COVID-19 mortality patients, 6 early COVID-19 mortality patients, and 10 organ donor controls using a 760-gene panel encompassing transcripts involved in both immune cell-tissue interactions and lung tissue homeostasis. Control samples were collected prior to 2019 from brain-dead organ donors over age 40 (median, 60.5; range 42–92 years) and did not show evidence of viral respiratory infection, pulmonary cause of death, or histological patterns of ALI (data not shown).

**Figure 2:**
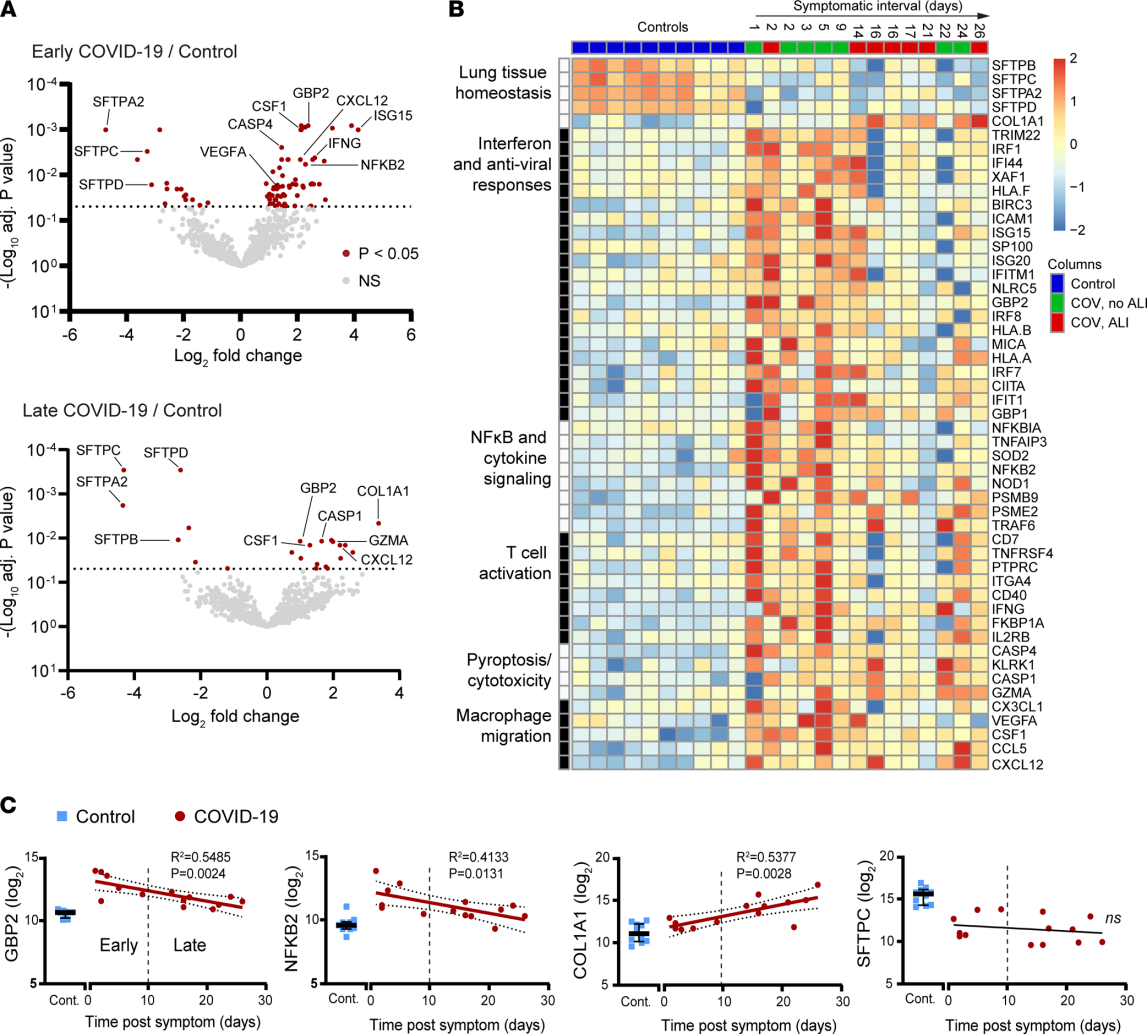
Gene expression profiles in early and late COVID-19 mortality. (**A**) Bowtie plots showing gene expression log_2_ fold change plotted against –log_10_-adjusted *P* value for comparison of the early (top left, *n* = 6) and late COVID-19 mortality cases (bottom left, *n* = 8) versus uninfected controls (*n* = 10). Red dots correspond to gene expression changes for the indicated comparison with adjusted *P* < 0.05. *P* values were adjusted for multiple-hypothesis testing using the Benjamini-Hochberg method. (**B**) Heatmap depicting the normalized and scaled transcript levels across COVID-19 cases (ordered by symptomatic interval) and controls for all the significantly altered transcripts falling into the indicated functional categories. (**C**) Dot plots depicting log_2_-normalized counts of the indicated transcripts. Controls are shown to the left (blue squares, *n* = 10), and COVID-19 cases are plotted against symptomatic interval (red dots, *n* = 14). The best-fit line with 95% confidence bands and *R*^2^ and *P* values were calculated using simple linear regression analysis. Error bars show median and interquartile range.

In comparison with control lungs, early mortality COVID-19 cases showed 59 upregulated transcripts and 16 downregulated transcripts (Figure 2A and Supplemental Figure 1) using the cutoff adjusted *P* value < 0.05 and log fold change (FC) ≥ 1. Upregulated genes in early COVID-19 mortality included many involved with interferon signaling (e.g., *GBP2*, *ISG15*), the NF-κB pathway (e.g., *NFKB2*, *NFKBIA*), and TNF-α and IL-1 signaling (e.g., *TANK*, *NOD1*, *RELA*). Late COVID-19 mortality showed 16 upregulated transcripts and 8 downregulated transcripts (Figure 2A and Supplemental Figure 2). Upregulated genes in late COVID-19 mortality included those involved in pyroptosis (30) (e.g., *GZMA*, *CASP1*) and fibrosis (*COL1A1*). Notably, both the early and late mortality cases showed marked coordinate downregulation of genes associated with T2AE cells and encoding pulmonary surfactant proteins (*SFTA2*, *SFTPB*, *SFTPC*, *SFTPD*) (Figure 2, A and B).

In gene set enrichment analysis (31–33), transcripts upregulated in the early COVID-19 mortality cases showed the greatest enrichment for Gene Ontology (GO) terms related to antiviral immune responses, including interferon-gamma-mediated signaling pathway (GO:0060333, 15/68 transcripts, early vs. 4/68 transcripts, late), type I interferon signaling pathway (GO:0060337, 14/65 transcripts, early vs. 4/65 transcripts, late), and defense response to virus (GO:0051607, 10/133 transcripts, early vs. 2/133 transcripts, late). Upregulated transcripts in early and late COVID-19 mortality were both enriched for GO terms related to macrophage differentiation and migration (Supplemental Figure 3).

Most of the interferon and NF-κB pathway genes that were found to be increased in early COVID-19 mortality showed decreased expression in the late mortality cases (Figure 2B). Analysis of the relationship between gene expression and the symptomatic interval prior to death across all the COVID-19 mortality cases identified 72 transcripts that correlated (adjusted *P* value < 0.1) with symptomatic interval (Supplemental Table 2). Genes that were found to be upregulated in the early COVID-19 mortality cases and involved with the interferon (*ISG20*, *IRF1*, *GBP2*) and NF-κB (*NFKB2*, *NFKBIA*, *TNFAIP3*) pathways showed linear decline with longer symptomatic interval (Figure 2C). In contrast, the fibrosis-related transcript *COL1A1* showed linear increase with symptomatic interval (Figure 2C) consistent with a fibrotic response to prolonged lung injury. In contrast, the surfactant transcripts remained profoundly suppressed across the full range of symptomatic intervals (Figure 2C). These results show that lung gene signatures of T2AE cell dysfunction define all fatal COVID-19 cases whereas the gene signatures of inflammation and antiviral responses are highly dependent on timing and stage of disease.

### Patterns of alveolar epithelial cell loss in fatal COVID-19.

Given the central role of alveolar epithelial cells in pulmonary surfactant production, gas exchange, and tissue repair (20, 27), we performed quantitative alveolar epithelial cell profiling on COVID-19 cases and uninfected controls. All alveolar epithelial cells express high levels of nuclear transcription termination factor 1 (TTF-1), a member of the Nkx2 family of homeodomain-containing transcription factors and a master regulator of pulmonary epithelial cell differentiation (34, 35). T2AE cells coexpress nuclear TTF-1 and cytoplasmic Napsin-A, a key enzyme in pulmonary surfactant production (Supplemental Figure 4A) (36). T1AE cells express nuclear TTF-1 without cytoplasmic Napsin-A (37–40). Using flow cytometry, we further validated a TTF-1 and Napsin-A dual-labeling strategy for classifying alveolar epithelial cells, by using the additional T2AE cell–specific marker, HTII-280 (41) (Supplemental Figure 4B).

**Figure 3:**
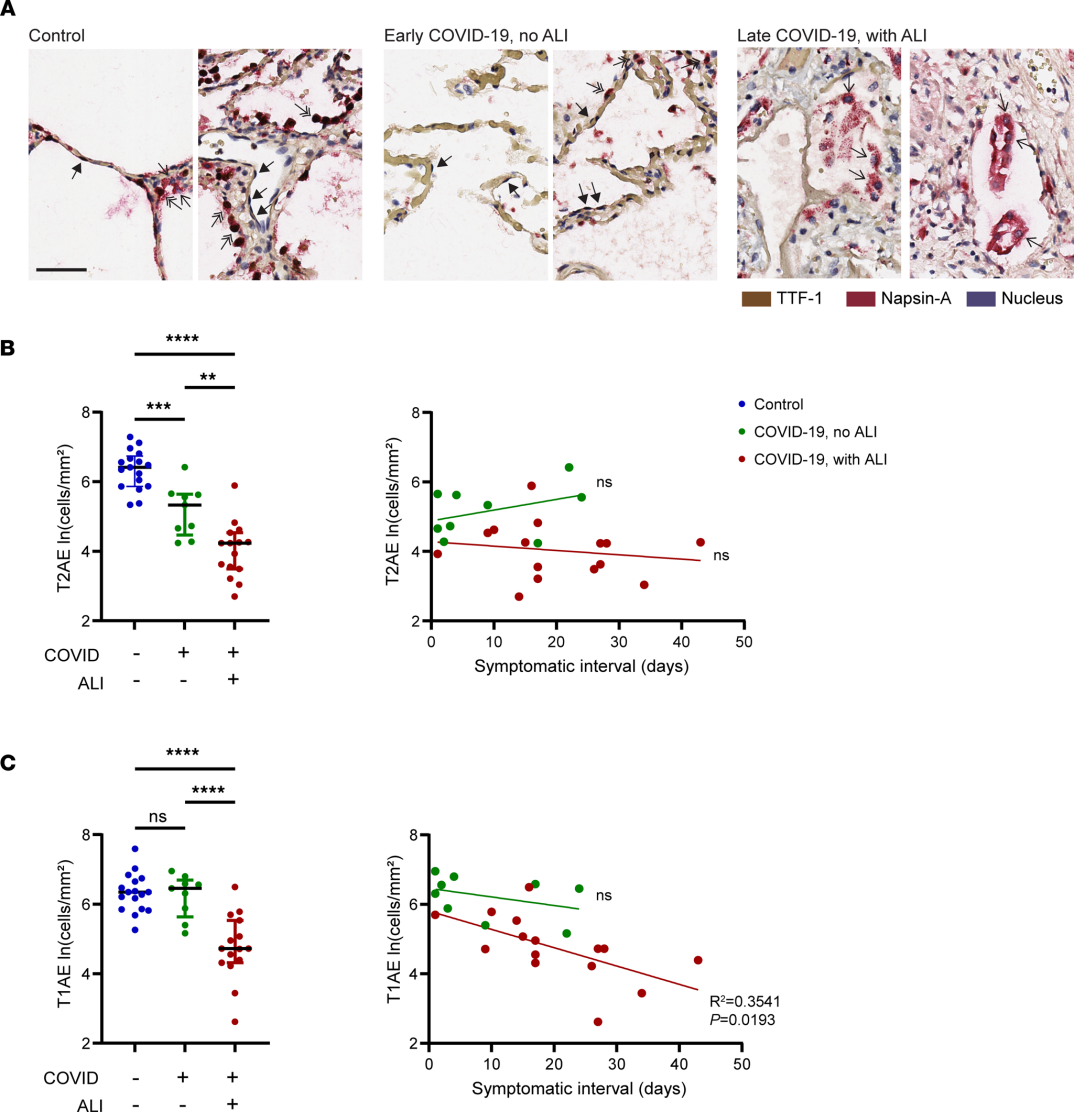
Distinct profiles of alveolar epithelial cell loss associated with lung tissue pathology in fatal COVID-19. (**A**) Dual-chromogen staining in alveolar lungs for nuclear TTF-1 (brown) and cytoplasmic Napsin-A (red) with hematoxylin counterstain (blue). Shown are representative original magnification 40× fields from 2 uninfected controls (left), early COVID-19 mortality cases without ALI (middle), and late COVID-19 mortality cases with ALI (right). (**B** and **C**) Dot plots depicting type 2 alveolar epithelial cell density (T2AE, TTF-1^+^Napsin-A^+^) (**B**, left) and type 1 alveolar epithelial cell density (T1AE, TTF-1^+^Napsin-A^–^) (**C**, left) in the controls (*n* = 17) compared with the COVID-19 mortality cases with (*n* = 15) and without (*n* = 9) ALI (indicated on the *x* axis). Also shown are the T2AE (**B**, right) and T1AE (**C**, right) cell densities plotted against the symptomatic interval. The best-fit line with 95% confidence bands and *R*^2^ and *P* values were calculated using simple linear regression analysis. Black scale bar: 50 μm. Solid arrow, T1AE cells; double arrow, T2AE cells; single arrow, macrophages. Error bars show median and interquartile range. **P < 0.01, ***P < 0.001, ****P < 0.0001.

We used dual chromogen immunohistochemistry to stain lung tissue sections of the COVID-19 cases and controls for TTF-1 (brown) and Napsin-A (red) with hematoxylin nuclear counterstain (Figure 3A). Cells were classified as T2AE based on nuclear TTF-1^+^ and cytoplasmic Napsin-A^+^ staining (TTF-1^+^Napsin-A^+^), and these cells showed characteristic cuboidal morphology and localization along the alveolar airspaces in the control lung (Figure 3A, double arrows) (20, 41). Cells were classified as T1AE based on nuclear TTF-1^+^ and cytoplasmic Napsin-A^–^ staining (TTF-1^+^Napsin-A^–^), and these cells showed characteristic thin nuclei and cytoplasm lining the alveoli (Figure 3A, solid arrows) (39, 40). The cells with Napsin-A^+^ cytoplasm and TTF-1^–^ nuclei (Figure 3A, single arrows) comprised mostly macrophages, and these cells were not quantified in this analysis (37).

Quantification of the alveolar epithelial cells revealed selective loss of T2AE cells in the COVID-19 mortality cases that lacked histologically apparent ALI, with further decline in T2AE cells associated with presence of ALI (Figure 3B, left). Marked depletion of T2AE cells was observed across the full range of symptomatic intervals (Figure 3B, right). In contrast, we observed significant loss of T1AE cells only in those cases with ALI (Figure 3C, left). Whereas T1AE cells remained intact in the early mortality cases, T1AE density showed linear decrease with increasing symptomatic interval specifically in those cases with ALI (Figure 3C, right). These data show that T2AE cell loss is a defining characteristic of fatal COVID-19 even at early stages before ALI is histologically apparent and that ALI development is associated with further loss of T2AE cells and with the time-dependent loss of T1AE cells.

### Aging drives selective T2AE cell loss in the uninfected lung.

**Figure 4:**
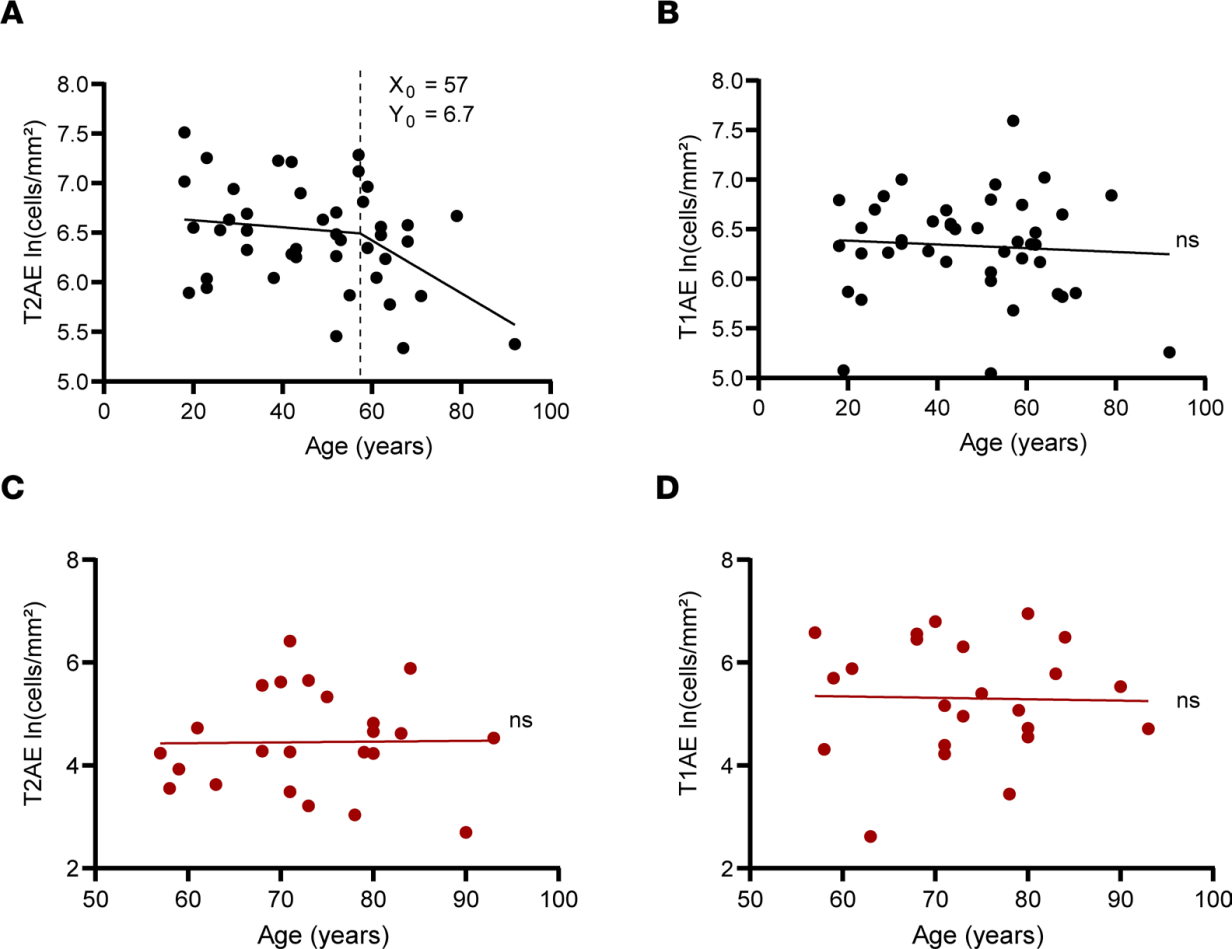
Age-associated changes in lung alveolar epithelial cells in uninfected organ donors and COVID-19 mortality cases. Dot plots depicting T2AE (**A**) and T1AE (**B**) cell density in uninfected organ donors (black dots, *n* = 43) plotted against donor age. Also shown are dot plots depicting T2AE (**C**) and T1AE (**D**) cell density in the COVID-19 cases (*n* = 24, red dots). The best-fit line and *P* values were calculated using simple linear regression analysis. The X_0_ and Y_0_ values for the age versus T2AE cell density plot were calculated using segmental linear regression where appropriate.

Given the key role of alveolar epithelial cells in respiratory function and lung repair, we examined whether patient age — the dominant risk factor for COVID-19 severity and mortality — is a predictor for alveolar epithelial cell density. We first examined the baseline state of alveolar epithelium in the uninfected lung from 43 organ donors with an age range of 18–92 years. We observed reduced T2AE cell density in lungs from the organ donors with increasing age (Figure 4A). Segmental linear regression analysis revealed an inflection point around 57 ± 11 (X_0_ ± SEM) years of age (Figure 4A), after which age-related T2AE cell decline was accelerated. In multiple linear regression analysis of the uninfected cohort, age was found to be a significant predictor of lower T2AE cell density after adjustment for smoking status and presence of underlying lung disease (Supplemental Table 3). In contrast, no relationship was observed between age and T1AE cell density (Figure 4B and Supplemental Table 3). In the COVID-19 mortality cases, T2AE cell density was markedly reduced (Figure 3B) in all cases; however, age was found not to be a significant predictor of T2AE (Figure 4C) or T1AE (Figure 4D) cell density. These findings show that lower baseline T2AE cell density is associated with increased age, which may increase susceptibility of alveolar epithelium to damage in COVID-19.

### Enhanced lymphocyte cytotoxicity and CD4^+^ macrophage infiltration in fatal COVID-19.

**Figure 5:**
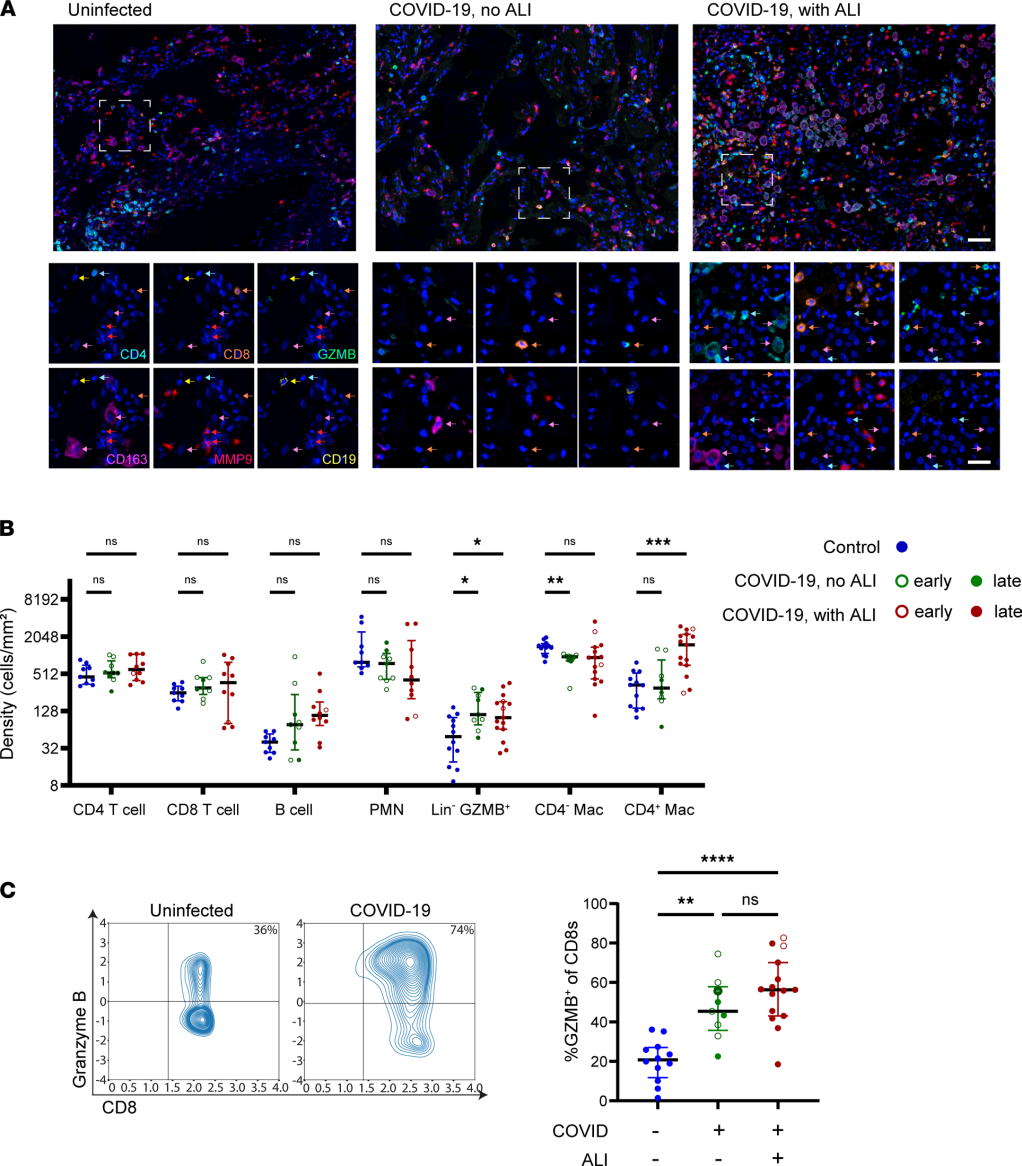
Patterns of immune cell infiltration associated with lung tissue pathology in fatal COVID-19. (**A**) Representative original magnification 20× fields of 7-color multiplex staining with single markers shown for the boxed magnified field, of alveolar lung tissue from uninfected controls (left), early COVID-19 mortality cases without ALI (middle), and late COVID-19 mortality cases with ALI (right). (**B**) Lung tissue density of immune cell subsets defined based on expression of the 6 immune lineage markers for each of the controls (*n* = 9–12, blue dots), COVID-19 mortality cases without ALI (*n* = 9, green dots), and COVID-19 mortality cases with ALI (*n* = 10–15, red dots). *P* values were calculated using a mixed effects model 2-way ANOVA and Dunnett’s multiple comparisons test. (**C**) Representative contour plots depicting granzyme B (GZMB) expression plotted against CD8 expression (left) in the imaged CD8^+^ T cells of a control and COVID-19 mortality case with compiled data (right). For each case, mortality at early (unfilled dot, *n* = 8) or late (filled dot, *n* = 16) stage of disease is also indicated. *P* values were calculated using Welch’s ANOVA test and Dunnett’s multiple comparisons test. *P < 0.05, **P < 0.01, ***P < 0.001, ****P < 0.0001. Error bars show median and interquartile range. White scale bar, top images 50 μm; single marker images 25 μm. Arrow colors indicate lineages (magenta, macrophage; cyan, CD4^+^ T cell; orange CD8^+^ T cell; red, neutrophil; yellow, B cell).

To elucidate factors associated with the local lung immune response that may drive alveolar epithelial injury in COVID-19, we profiled the major immune lineages in lung tissue of the COVID-19 cases and controls. Representative sections from each lung sample were stained using a 6-color immune lineage panel with markers to delineate B cells (CD19), T cells (CD4 and CD8), neutrophils (MMP9), macrophages (CD163), and the cytotoxic effector molecule granzyme B (GZMB) (Figure 5A). Image analysis was performed on 20–50 original magnification 20× fields of alveolar lung per person (1354 fields total), and classification into immune cell lineages was performed on each cell using information from all 6 markers with a trainable machine learning algorithm (inForm 2.3, PerkinElmer) (see Methods and Supplemental Figure 5A).

Quantification of the immune cell density in lung tissue revealed no significant differences in the major lymphocyte lineages or neutrophils (Polymorphonuclear neutrophil, PMN) in the COVID-19 mortality cases (Figure 5B). However, we did observe increased GZMB expression in the CD8^+^ T cells (Figure 5, A and C) as well as increased density of Lin^–^GZMB^+^ cytotoxic cells in all the COVID-19 mortality cases (Figure 5B). These cytotoxic cells were often found adjacent to or within the alveolar wall (Figure 5A, middle and right) and were increased similarly across early and late stages of COVID-19 and lung injury patterns (Figure 5, B and C). Thus, alveolar infiltration by cytotoxic lymphocytes occurs in early stages of COVID-19 prior to development of ALI.

With the development of ALI, we observed marked changes in the lung macrophage population. Whereas uninfected lung tissue contains a predominant CD163^+^ macrophage population that is negative for the other lineage markers, we observed increased density of CD4^+^CD163^+^ macrophages in the COVID-19 cases with ALI (Figure 5, A and B, and Supplemental Figure 5B). These CD4^+^ macrophages were predominantly round to oval, lacking in processes with high cytoplasm to nuclear ratio, consistent with the in situ appearance of alveolar rather than interstitial macrophages (Figure 5A, right) (42). In addition, we observed modestly but significantly reduced density of the CD4^–^CD163^+^ macrophages in the COVID-19 cases without ALI (Figure 5B). These results show that ALI in COVID-19 is associated with infiltration of CD4^+^ macrophages.

### Increased T cell cytotoxicity correlates with alveolar epithelial damage in COVID-19.

**Figure 6:**
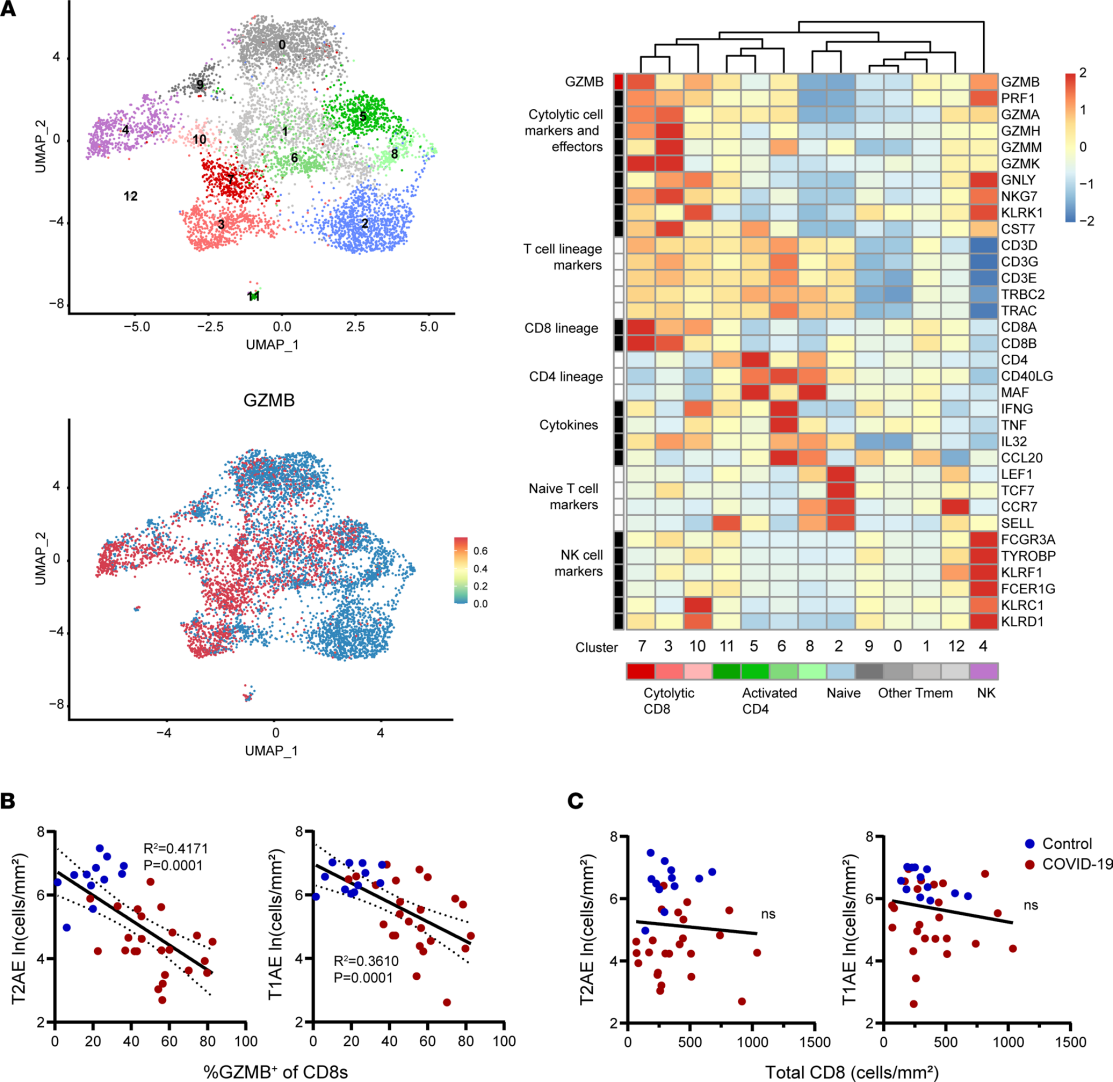
Correlation of lung T cell cytotoxicity with alveolar epithelial cell loss in fatal COVID-19. (**A**) Uniform manifold approximation and projection (UMAP) embeddings of total T/NK cells obtained from airways of 3 patients with COVID-19 (top left) with a feature plot showing normalized expression of GZMB (bottom left). Representative marker genes for each cluster are shown in the normalized and scaled heatmap to the right, with color bars corresponding to position on the UMAP. (**B**) The percentage GZMB^+^ cells within the CD8^+^ T cell subset in lungs and (**C**) the overall density of all CD8^+^ T cells is plotted against the density of lung T2AE cells (left) and T1AE cells (right) from all COVID-19 cases (*n* = 24) and controls (*n* = 12). The best-fit line with 95% confidence bands and *R*^2^ and *P* values were calculated using simple linear regression analysis.

To better define how cytotoxic immune cells may contribute to alveolar epithelial damage, we performed reanalysis of the T and NK cell transcriptome signatures from a previously published single-cell RNA-Seq (scRNA-Seq) data set of bronchoalveolar (BAL) washings from 3 patients intubated with severe COVID-19 (serially sampled at days 1–6, 8–10, and 19–22 after symptom onset) (22). After selection of highly variable features and clustering (Figure 6A, top left), *GZMB* expression was detected predominately in a subset of CD8^+^ T cells comprising 3 clusters (clusters 3, 7, and 10). We found that the CD8^+^ T cells expressing the highest levels of *GZMB* also had high coexpression of multiple transcripts encoding cytotoxicity molecules (e.g., *PRF1*, *GZMA*, *GZMK*, *GNLY*, *CST7*) (Figure 6A, right) in contrast with the other subsets. Expression of these same cytolytic markers is also shared by a single cluster of NK cells (cluster 4) defined by high expression of NK lineage markers and lack of T cell lineage markers (Figure 6A, right). These results show that the GZMB^+^CD8^+^ cells in COVID-19 lung tissue likely coexpress a broad range of cytotoxic effector molecules, which may contribute to local tissue damage.

We further defined the relationship between T cell cytotoxicity and the density of TTF-1^+^ alveolar epithelial cells. The percentage of CD8^+^ T cells expressing GZMB was significantly correlated with greater depletion of T1AE cells and T2AE cells across all the COVID-19 cases (Figure 6B). In contrast, overall CD8^+^ T cell density (encompassing GZMB^+^ and GZMB^–^) was found not to be significantly correlated with alveolar epithelial cells (Figure 6C). In a multivariable linear regression model of the COVID-19 cases, the expression of GZMB in the CD8^+^ T cells was found to be a significant predictor of lower alveolar epithelial cell density after adjustment for confounding factors including age, presence of ALI, and length of symptomatic interval (Supplemental Table 4). These results show that increased T cell cytotoxicity correlates with increased extent of alveolar epithelial damage in fatal COVID-19.

### CD4^+^ macrophages express gene signatures of T cell activation and tissue inflammation.

**Figure 7:**
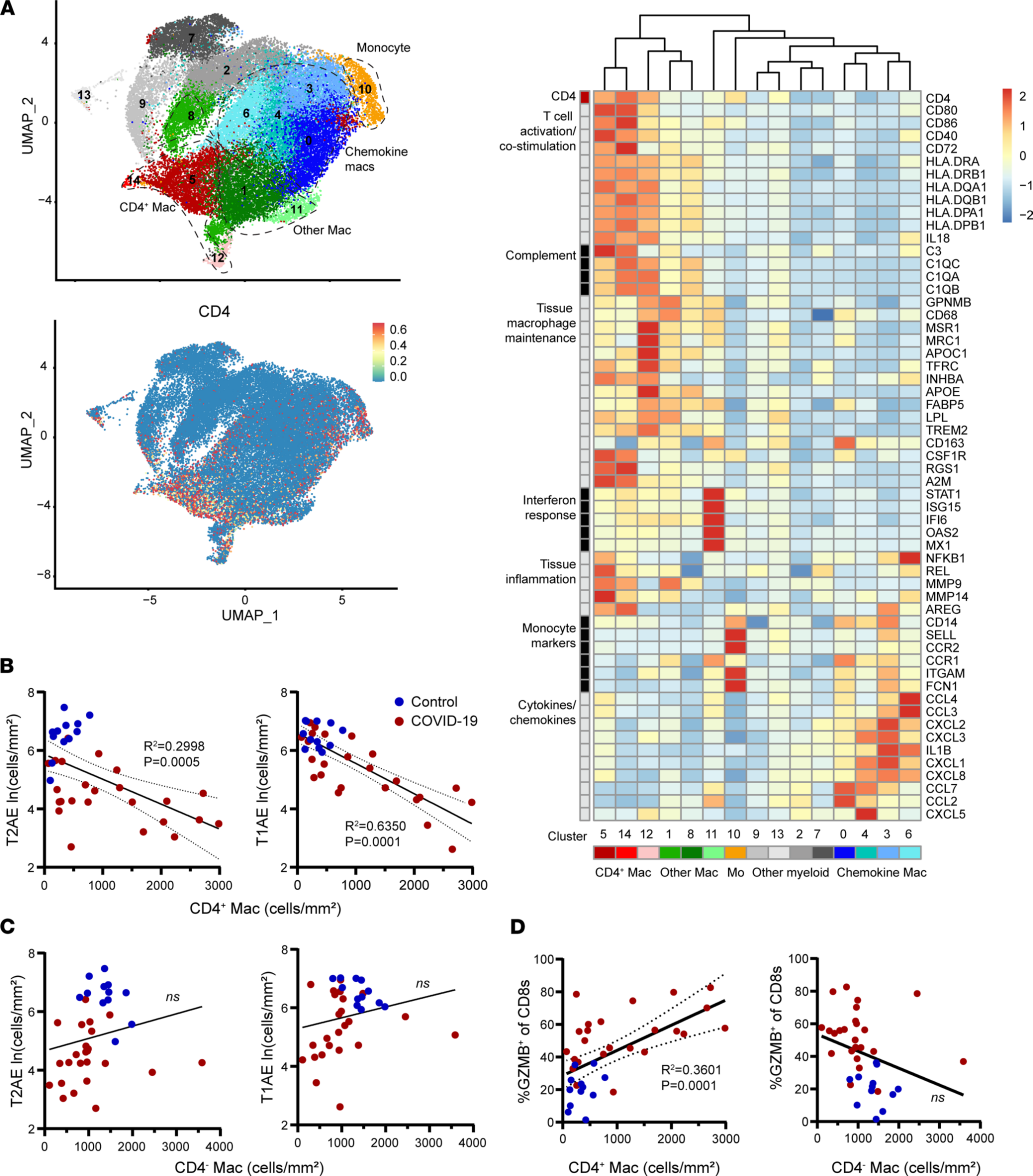
Correlation of CD4^+^ macrophages with T cell cytotoxicity and alveolar epithelial cell loss in fatal COVID-19. (**A**) UMAP embeddings of total monocytes (Mo) and macrophages (Mac) from airways of 3 patients with COVID-19 (top left). Feature plot shows normalized expression of CD4 (bottom left). Representative marker genes for each cluster are shown in the normalized and scaled heatmap on the right with color bars corresponding to position on the UMAP. (**B** and **C**) The density of CD4^+^ macrophages (**B**) and CD4^–^ macrophages (**C**) in lung tissue is plotted against the density of lung T2AE cells (left) and T1AE cells (right). (**D**) The density of CD4^+^ macrophages (left) and CD4^–^ macrophages (right) is plotted against the percentage of GZMB^+^ cells in the CD8^+^ T cell subset for all COVID-19 cases (*n* = 24) and controls (*n* = 12). The best-fit line with 95% confidence bands and *R*^2^ and *P* values were calculated using simple linear regression analysis.

The strong association of CD4^+^ macrophages with ALI in COVID-19 prompted us to assess myeloid cell transcriptomes for distinct subsets associated with CD4 expression. We performed reanalysis of the previously published myeloid cell transcriptomes in the scRNA-Seq data set of BAL washings from patients with severe COVID-19 (22). After selection of highly variable features in the myeloid data set and clustering, *CD4* expression was detected predominately in a transcriptionally distinct subset of the BAL myeloid cells corresponding to 3 clusters (cluster 5, 12, and 14; Figure 7A). Analysis of these clusters revealed high expression of core macrophage lineage genes, including *CD68* and *CSF1R*, in addition to scavenger receptor transcripts associated with alveolar macrophages (the genes *MRC1* and *MSR1*, encoding CD206 and CD204) (43). In contrast with interstitial macrophages, which express high levels of CD169 and low levels of CD11c and produce IL-10, the CD4^+^ macrophages expressed relatively high levels of CD11c transcript (*ITGAX*), low levels of CD169 transcript (*SIGLEC1*), and low *IL10* transcript (Supplemental Figure 6) (42, 44).

Notably, the CD4^+^ macrophages also showed distinctly high expression of several transcripts associated with T cell activation, including all components of MHC class II (*HLA-DRA*, *HLA-DRB1*, *HLA-DQA1*, *HLA-DQB1*, *HLA-DPA1*, and *HLA-DPB1*), as well as costimulation molecules (*CD80*, *CD86*, *CD40*, and *CD72*) and transcripts associated with inflammation, tissue damage, and fibrosis (*NFKB1*, *MMP9*, *MMP14*, and *AREG*) (45, 46) (Figure 7A). High MHC class II expression is not typical of steady-state resident alveolar macrophages (44) but has been reported in monocyte-derived alveolar macrophages, which are recruited in the setting of lung inflammation (24, 47, 48). The CD4^+^ macrophages in COVID-19 also expressed several other markers of monocyte-derived alveolar macrophages, including the *APOE* transcript and genes encoding the complement component C1Q (24, 47, 48).

Previous studies have identified high levels of chemokine expression as well as IL-1β expression in the lung macrophages of patients with severe COVID-19 (22, 23). In this data set, the highest expression of chemokines and IL-1β was found in transcriptionally distinct clusters (0, 3, 4, and 6) separate from the CD4^+^ macrophage subset (Figure 7A). The chemokine- and IL-1β–expressing macrophage subsets were found to express the lowest levels of *CD4* and T cell activation genes (Figure 7A). These results show that *CD4* transcript corresponds with macrophage subsets expressing high levels of T cell activation genes and are distinct from the chemokine-producing macrophage subsets also infiltrating lungs of patients with severe COVID-19.

### CD4^+^ macrophages predict epithelial cell loss and lymphocyte cytotoxicity.

The distinctly high coexpression of T cell activation molecules with proinflammatory mediators in the CD4^+^ macrophage population prompted further analysis of how these cells correlate with the local epithelial and immune cells in the lung. In a linear regression analysis, we observed increased CD4^+^ macrophage density in COVID-19 autopsy samples to be strongly correlated with decreased density of alveolar epithelial cells (Figure 7B). Multivariable analysis of the COVID-19 cases showed that higher CD4^+^ macrophage density and lung macrophage CD4 expression level both remained significant predictors of lower alveolar epithelial cell density even after adjustment for the presence of ALI (Supplemental Tables 5 and 6). In contrast, the CD4^–^ macrophage density did not correlate with alveolar epithelial cells (Figure 7C). In addition, we found the CD4^+^ macrophage density to be positively correlated with GZMB expression in the CD8^+^ T cells whereas the lung CD4^–^ macrophages density was not found to correlate with GZMB expression in CD8^+^ T cells (Figure 7D). These results show that CD4^+^ macrophages, but not CD4^–^ macrophages, are associated with alveolar epithelial dysfunction and lymphocyte cytotoxicity in fatal COVID-19.

## Discussion

SARS-CoV-2 infection is uniquely destructive to the lung’s alveolar gas exchange interface, particularly in older individuals. Understanding the mechanisms underlying alveolar damage and age-related risk in COVID-19 is required to improve patient outcomes and for addressing future respiratory pandemic threats. Due to the severity of COVID-19, strain on emergency medical and hospital resources, and disparities in health care access, many patients with COVID-19 die before supportive care can be established or after prolonged hospitalization due to refractory disease (4). Therefore, autopsy studies have played a pivotal role in defining mechanisms of COVID-19 pathogenesis. Previous autopsy studies have identified distinct immunopathological stages in fatal COVID-19 and defined the transcriptome signatures of lung epithelial and immune dysfunction at single-cell resolution (12, 18, 23, 24, 29, 49). With this study, we have elucidated potentially novel facets of the immunopathological processes driving alveolar destruction in fatal COVID-19 using coordinated analysis of epithelial and immune cell subsets in lung tissue from early and late COVID-19 mortality cases and uninfected organ donors.

Although previous studies have described T2AE and T1AE cell loss and dysfunction in fatal cases of COVID-19 (23, 49) and in other severe lung diseases (27), this study provides evidence that selective T2AE cell loss occurs in early stages of COVID-19 prior to development of ALI (Figure 2C and Figure 3B) and thus may play a primary role in respiratory failure. Consistent with our results in tissue, recent studies in blood have shown that biomarkers of T2AE cell damage are increased in the earliest stages of severe COVID-19 (50). Loss of T2AE cells may promote alveolar collapse and prevent alveolar repair (20, 21). Surfactant is primarily produced by T2AE cells and is required to maintain alveolar surface tension (20, 21). In murine models, alveolar surfactant loss, by itself, can disrupt pulmonary gas exchange and cause hypoxemia (51, 52). Thus, acute loss of surfactant may contribute to “silent hypoxia” in COVID-19 before ALI, inflammation, and edema are histologically or radiographically apparent (11, 14–16, 53). Also, T2AE cells have progenitor activity that is required to regenerate damaged alveolar epithelium (20, 21, 27), and thus T2AE cell loss in early-stage COVID-19 may contribute to progressive depletion of T1AE cells (Figure 3C) and inability to recover alveolar function.

Lower T2AE cell density associated with increased age in the uninfected cohort (Figure 4A) may reflect diminished functional reserve and regenerative capacity of alveolar epithelium (20, 21, 27). Thus, an impaired T2AE cell baseline may underlie susceptibility of older individuals to developing severe COVID-19. In the setting of fatal COVID-19 and evolving ALI, alveolar epithelial cell depletion is primarily correlated with cytotoxic T cells and CD4^+^ macrophages (Figure 6B and Figure 7B), rather than age (Figure 4C). Thus, immune-mediated damage may exert dominant effects on alveolar epithelial cells once severe disease is established in the absence of significant regenerative responses.

Widespread damage to the alveolar epithelium in COVID-19 is probably mediated by the tissue immune response (14, 24, 29). Most studies investigating immune mechanisms of lung injury in COVID-19 have focused on the role of macrophages, the predominant immune subset in lungs (12, 14, 18, 22). Our study reveals that cytotoxic lymphocytes are increased in early COVID-19 prior to macrophage infiltration and development of ALI (Figure 5, B and C). Although a previous study has shown increased concentration of the cytotoxic effector molecules GZMB and perforin in airway supernatant of patients with COVID-19 (22), we believe our study is the first to demonstrate the marked induction of GZMB in situ within the CD8^+^ T cell compartment in early COVID-19 (Figure 5) and the correlation of lymphocyte cytotoxicity with alveolar epithelial cell loss (Figure 6B and Supplemental Table 4). Our analysis of scRNA-Seq data from airway immune cells of patients with COVID-19 (Figure 6A) showed that the highest *GZMB* expression corresponded to broadly cytotoxic gene signatures in CD8^+^ T cells, supporting a role for CD8^+^ T cell cytotoxicity in alveolar epithelial destruction.

Increased lung macrophage density in COVID-19 is closely associated with ALI (12, 14, 18, 22), and scRNA-Seq studies show proinflammatory macrophage gene expression signatures in COVID-19 with distinctly high expression of IL-1β and chemokines that may propagate lung inflammation (12, 18, 23, 24). Our study is the first to our knowledge to describe a distinct CD4-expressing macrophage subset that becomes predominant specifically in the COVID-19 cases with ALI (Figure 5B). Previously, CD4 expression has been described on tissue-resident macrophages in murine intestine at steady state, and CD4 expression on human monocytes can mediate macrophage activation and differentiation upon ligation (54, 55). The COVID-19–associated CD4^+^ macrophages have morphologic characteristics of alveolar macrophages (Figure 5A) (42, 44). However, their transcriptome signature and CD4 expression suggest that they may make up a subset of monocyte-derived, rather than tissue-resident, alveolar macrophages that are recruited due to lung inflammation (24, 47, 48). Compared with other pulmonary myeloid cell populations in COVID-19, the transcriptome of the CD4^+^ macrophage subset suggests low cytokine production but high capacity to activate local T cells through costimulation and antigen presentation (Figure 7A). The association of increased CD4^+^ macrophage density with higher CD8^+^ T cell cytotoxicity and increased alveolar epithelial cell loss (Figure 7, B and D, and Supplemental Table 5) suggests a potential role for CD4^+^ macrophages in orchestrating alveolar damage through T cell interactions, although these correlations do not definitively establish causal relationships between these immune cells and lung damage.

In summary, our study provides an integrative perspective of COVID-19 immunopathology at the lung’s gas exchange interface. It has identified key unifying characteristics across the heterogeneous infection course and patterns of lung tissue injury seen in COVID-19 mortality, identified age-related changes in alveolar epithelium of uninfected individuals that may contribute to COVID-19 risk, and defined dynamic interrelationships between alveolar epithelial states and immune cells at different stages of lethal infection. Together, these findings advance our understanding of the immune and epithelial factors associated with respiratory failure in COVID-19 and age-related COVID-19 risk. Our results provide key insights into potential immune mechanisms of alveolar damage and may inform the design of therapeutic strategies that more specifically target the immunopathological mechanisms operative at distinct stages of pulmonary viral infection.

## Methods

### COVID-19 case definition and sample collection.

The study was approved by the institutional review board of Columbia University Irving Medical Center (CUIMC) and conducted according to institutional review board (IRB) requirements. The analysis of lung tissue samples was completed for 24 SARS-CoV-2 autopsies confirmed with pre- or postmortem reverse transcription PCR. Autopsies were performed in Columbia University NewYork-Presbyterian Hospital, in accordance with guidelines set forth by the College of American Pathologists and recommendations provided by the US Centers for Disease Control and Prevention. Autopsies were completed using the Virchow technique, in a negative-pressure autopsy suite with appropriate personal protective equipment, including N-95 masks; eye protection; and disposable scrub caps, gowns, gloves, and rubber boots. The lungs were dissected and fixed in formalin by instillation of fixative solutions. Sections were taken from grossly or radiographically identified abnormal regions from each lung lobe. At least 1 section from each lung lobe was taken and submitted in standard tissue cassettes. Tissue was processed and embedded in paraffin.

### Collection and processing of control samples.

Control, uninfected lung tissues were obtained from deceased organ donors prior to 2019 as part of organ acquisition for clinical transplantation through an approved protocol and material transfer agreement with LiveOnNY as described previously (56, 57). Donors did not have pulmonary cause of death and were free of cancer and chronic diseases; seronegative for hepatitis B, hepatitis C, and HIV; and negative for SARS-CoV-2 by PCR. Use of organ donor tissues does not qualify as “human subjects” research, as confirmed by the Columbia University IRB, as tissue samples were obtained from brain-dead (deceased) individuals. Lung tissue sections (<5 mm thickness) were fixed in zinc-buffered formalin (Anatech Ltd.) for 48–72 hours and embedded in paraffin for long-term storage. Donor medical history obtained from the next of kin was provided through the organ procurement organization.

### Histological analysis of the cases and controls and sample selection.

H&E staining of the lung sections from all cases and controls was comprehensively analyzed by a pulmonary pathologist. The main histological classification was based on presence or absence of ALI. ALI was diagnosed by presence of DAD or fibrin, affecting more than 1 slide and at least 5% of the slide area (11, 28). Presence of vascular congestion and hemangiomatosis-like change was also analyzed (11).

One representative tissue block was selected from each case for RNA and image analysis based on lack of autolysis, presence of pathological changes representative of the overall case definition, and patterns of predominately alveolar lung without disproportionate vessels, large airway components, or nonpulmonary tissue.

### RNA extraction and analysis for cases and controls.

The RNA was extracted from a 20 μm thick tissue section using the RNeasy FFPE Kit (Qiagen) according to the manufacturer’s instructions by the Molecular Pathology Shared Resource core facility at CUIMC. The RNA concentration and size distribution for each sample was assessed using NanoDrop (Thermo Fisher Scientific) and Bioanalyzer (Agilent). RNA expression profiling was performed using nCounter Human Organ Transplant Panel (nanoString) to profile 770 genes in pathways critical for tissue homeostasis and immune-mediated tissue damage. The RNA samples passing quality and concentration standards were run in 2 batches each, including standards for batch calibration. Input RNA amounts to the nanoString assay were adjusted for RNA integrity as recommended by the manufacturer and were hybridized to target-specific probes and controls in a single tube for 20 hours at 65°C using 100–900 ng of RNA. Target-probe complexes were purified and immobilized on the nCounter prep station. Using the nCounter detection analyzer (nanoString), digital counts for each target RNA were acquired. Finally, nSolver software (nanoString) was used for batch calibration and normalization with housekeeping genes (*G6PD*, *OAZ1*, *ABCF1*, *TBP*, *POLR2A*, *NRDE2*, *GUSB*, *TBC1D10B*, *SDHA*, *UBB*, *PPIA*, *STK11IP*). The nanoString assay includes RNA spike ins, labeled A–F in decreasing order of concentration, with positive spike in F (POS_F) in the raw data accounting for the lower limit of detection. Thus, transcripts with undetectable copy number are excluded (normalized to 0).

Differential gene expression analysis was performed using nCounter Advanced Analysis Software (nanoString, version 2.0.134) with comparison of the early (≤10 days symptomatic interval, *n* = 8) and late (≥10 days symptomatic interval, *n* = 6) mortality COVID-19, reference to a baseline of uninfected controls (*n* = 10), and *P* values adjusted using the Benjamini-Hochberg method of estimating FDRs. Differentially expressed transcripts were identified using an FDR-adjusted *P* value cutoff of 0.05. Pathway analysis and calculation of directed global significance scores for each pathway was performed using the Gene Set Analysis function of the nCounter Advanced Analysis Software. Heatmaps of directed global significance scores and normalized expression gene values of differentially expressed genes were generated using the *pheatmap* function in R with clustering by rows.

### Staining for flow cytometry of alveolar epithelial lineage markers in lung tissue suspensions.

Single-cell suspensions of organ donor lung tissue were processed as previously described (56, 57). Cryopreserved samples of the lung suspensions were thawed, washed, and stained with the Zombie NIR (BioLegend) Fixable Viability Kit according to the manufacturer’s directions. Samples were then washed in cell staining buffer (PBS with 2% FBS) and stained with directly conjugated surface marker EpCAM-BV650, and unconjugated HTII-280 mouse monoclonal IgM (Terrace Biotech), at 4°C for 30 minutes, followed by incubation with FITC-conjugated anti–mouse IgM secondary (Jackson ImmunoResearch, Supplemental Table 7) in cell staining buffer. Following the surface stain, the samples were fixed and permeabilized for 1 hour at 4°C using a transcription factor staining buffer kit (Tonbo) followed by intracellular staining with directly conjugated TTF-1–PE (Miltenyi Biotec) and unconjugated napsin (Thermo Fisher Scientific) or surfactant protein C (Thermo Fisher Scientific) rabbit polyclonal and subsequent staining with APC-conjugated anti–rabbit IgG secondary antibody (Jackson ImmunoResearch, Supplemental Table 7). Single-cell fluorescence profiles were acquired from the cell suspension using the BD Fortessa flow cytometer (BD Biosciences).

### Dual chromogenic staining for TTF-1 and Napsin-A and alveolar epithelial cell profiling.

Representative tissue sections (5 μm) from each sample underwent heat-induced epitope retrieval with Bond Epitope Retrieval Solution 1 (Leica) and were subsequently stained with a prediluted multiplex TTF-1 (mouse monoclonal 8G7G3/1) + Napsin A (rabbit polyclonal) antibody reagent (Biocare). Staining and detection were performed using the Bond-III automated IHC stainer (Leica) and the ChromoPlex 1 Dual Detection system (Leica) (Supplemental Table 7). In this system an HRP- conjugated polyclonal anti-mouse IgG localizes the TTF-1–bound primary antibodies, and alkaline phosphatase–conjugated (AP-conjugated) polyclonal anti-rabbit IgG localizes Napsin-A–bound primary antibodies. The HRP chromogen substrate 3,3′-diaminobenizidine tetrahydrochloride hydrate (DAB) was used to label nuclear TTF-1 with brown color, and the AP chromogen substrate, Fast Red (Leica), was used to label cytoplasmic Napsin A with red color. Hematoxylin counterstaining was used to label all nuclei blue. After staining, all slides underwent rapid dehydration, were coverslipped with mounting media (Leica), and then were scanned and digitized using the SCN400 slide scanner (Leica).

Analysis of the whole slide scans was performed using HALO software implementing the multiplex IHC module (Indica Labs) at working analysis magnification of 30×. Fields for alveolar epithelial cell profiling were selected to include as much of the section comprising alveolar lung as possible, excluding the large airways and vessels. Field selection was performed by an individual following a protocol blinded as to experimental group. Each chromogen was defined by the distinct optical density (OD) values for the red, green, and blue components comprising its specific color. Values for separating and isolating the DAB (brown, TTF-1), Fast Red (red, Napsin-A), and hematoxylin (blue, nuclear) stains were optimized to be applicable across all stained slides. Nuclear segmentation was performed based on both the DAB and hematoxylin chromogens and optimized to be applicable across all stained slides and to detect the thin elongated nuclei characteristic of T1AE cells (39). The cytoplasmic compartment was sampled at a maximum 2 μm radius around the nuclei. TTF-1 nuclear positivity was defined by minimum average nuclear compartment DAB OD of 0.3 and Napsin-A cytoplasmic positivity was defined by minimum average cytoplasmic compartment Fast Red OD of 0.2. These thresholds were set based on examination of normal T2AE and T1AE cell staining in the uninfected control samples and on scatterplots generated of TTF-1 and Napsin A OD across all samples. For all fields selected for each sample, the T2AE cells were defined as nuclear TTF-1^+^ and cytoplasmic Napsin-A^+^, and T1AE cells were defined as nuclear TTF-1^+^ and cytoplasmic Napsin-A^–^. The TTF-1^–^ cells including macrophages expressing cytoplasmic Napsin-A were not quantified. The density of T2AE and T1AE cells was computed across the cellular area of lung tissue, excluding airspaces and vascular spaces, and natural log transformed for statistical analysis.

### Immunohistochemical staining for SARS-CoV-2 N protein.

Representative 5 μm FFPE lung tissue sections from each sample underwent heat-induced epitope retrieval with Bond Epitope Retrieval Solution 2 (Leica) and were subsequently stained with a monoclonal anti–SARS-CoV-2 N protein antibody (Sino Biological, clone 001). Staining and detection was performed using the Bond-III automated IHC stainer (Leica) with AP-conjugated polyclonal anti-rabbit IgG and the Fast Red substrate (Leica) to localize N protein–bound primary antibodies. After dehydration and application of coverslip and mounting media, the samples were scored as positive or negative by a pathologist following a blinded protocol.

### Multispectral staining and imaging of lung tissue.

Representative samples of lung tissue, 5 μm in thickness, were obtained from the cases and controls with selection based on lack of autolysis and presence of predominant patterns of alveolar lung without contaminating nonpulmonary tissue. Uninfected lung sections were obtained from deceased organ donors who were brain-dead due to nonpulmonary cause of death. None of the control samples showed pathological evidence of ALI.

Samples were fixed in 10% formalin (Anatech Ltd.) for 48 hours prior to dehydration and embedding in paraffin. These lung samples were sectioned at 5 mm thickness and stained using the Opal 7-Color Automated IHC Detection Kit (Akoya Biosciences) as previously described (58, 59). The multiplex panel included DAPI for nuclear counterstaining, CD4 (1:150 dilution), CD8 (1:600 dilution), CD163 (1:200 dilution), granzyme B (GZMB) (1:200 dilution), CD19 (1:50 dilution), and MMP9 (1:900 dilution) (see Supplemental Table 7 for clones and suppliers). Briefly, the Opal multiplex protocol involves multiple rounds of staining. Each round comprises incubation with primary antibody, followed by mouse/rabbit-specific HRP-conjugated secondary antibody, followed by Opal fluorescent substrate deposition. After each staining round the antibodies are stripped, thus enabling subsequent stains for distinct markers using mouse or rabbit primary antibodies. Single controls and an unstained slide were stained with each group of slides. After staining, the sections were mounted in Vectashield Hard Set mounting media (Vector Laboratories, catalog H1600) and stored at 4°C for up to 48 hours prior to image acquisition. Image acquisition was performed using the integrated Vectra 3 automated quantitative pathology imaging system (PerkinElmer). An initial whole slide scan at low magnification was performed prior to color deconvolution. Based on the whole slide scan, 20 to 50 fields evenly sampling the full surface of alveolar lung tissue across each slide were chosen by a pathologist following a protocol blinded to sample identity, for scanning at 20× original magnification (numerical aperture 0.75) (59). A total of 1354 original magnification 20× fields (each 0.67 mm × 0.5 mm) were analyzed from 24 COVID-19 cases and 12 uninfected controls. Images were analyzed using inForm software (Akoya Biosciences).

### In situ immune cell profiling in lung tissue.

Color deconvolution, cell segmentation, and phenotyping were performed using inForm software (Version 2.3, PerkinElmer/Akoya Biosciences) while blinded as to sample identity. Immune cell constituents within each tissue area were defined by the DAPI nuclear counterstain to define the nucleus of each cell, with each associated cytoplasm and membrane detected via presence of a specific stain (CD3, CD19, CD4, GZMB, MMP9, and/or CD163). Cell segmentation was adjusted as previously described to accurately locate all cells and minimize nuclear hypersegmentation and hyposegmentation (59). To ensure that cell phenotypes were not called by the software incorrectly, quality control was performed on the images from each patient, along with cell segmentation setting adjustments to correctly segregate membrane staining of adjacent cells and avoid erroneously grouping clusters of disparate cells. Cells were then phenotyped by training a machine learning classifier using inForm software. The classifier was trained based on expression of all 6 markers to identify monocyte/macrophage (CD163^+^, magenta cells), T cells (CD4^+^, cyan cells; and CD8^+^, orange cells), B cells (CD19^+^, yellow cells), neutrophils (MMP9^+^, red cells), and cytotoxic cells (GZMB^+^, green cells). After cell segmentation and phenotyping, outputs include the cell segmentation data summary providing densities and numbers of each cell type in the lung tissue areas and the full cell segmentation data file providing the *X* and *Y* coordinates of each phenotyped cell along with the fluorescence intensities.

For each sample, the full cell segmentation files were merged for each analyzed tissue area. The cell density of each phenotype was calculated as the number of cells per unit of cellular area, excluding airspaces and vascular spaces, and the density value was natural log transformed. Visualization of the classified cells was performed using UMAP based on the normalized fluorescence intensities for the 6 immune lineage markers and demonstrated cohesive clustering of the immune lineages (Supplemental Figure 4A). In total, 1354 original magnification 20× fields from 24 COVID-19 mortality cases and 12 controls were profiled, and 930,000 immune cells were analyzed. To generate the UMAP in Supplemental Figure 4A, the fluorescence intensities for each immune cell marker were transformed using arcsinh function from Python *numpy* library (60) using a cofactor calculated for each parameter using Otsu’s thresholding method (61) from the *scikit-image* toolbox from SciPy (62). Data from all immune cells from each condition were downsampled to display equal numbers of cells for each condition using RandomUnderSampler from the *imbalanced-learn* toolbox. The downsampled and transformed data set was used to run UMAP (63) for dimensionality reduction (n_neighbors = 15) using the 6 lineage markers. The data were projected in 2 dimensions using UMAP embeddings with colors indicating the immune phenotypes that were assigned using the machine learning classifier in inForm.

### Analysis of scRNA-Seq.

Focused analysis of myeloid cells and T/NK cells in COVID-19 was performed using an scRNA-Seq data set generated on the 10X Genomics Chromium platform of the cells in BAL lavage samples of 3 intubated COVID-19 patients (22) (https://www.covid19cellatlas.org/index.patient.html). The myeloid and T/NK cells were defined in the data set using an scRNA-Seq cell annotation method as previously described (22). Briefly, myeloid or T/NK cells were identified through positive and negative selection for highly expressed and specific lineage markers (*CD14*, *FCGR3A*, *CD163*) or T/NK markers (T cell: *CD3D*, *TRAC*, *TRBC1*, *TRBC2*, *TRDC*, *TRGC1*, *TRGC2*; NK cell: *NCAM1*) with 2 successive rounds of clustering for identification and removal of multiplets (22).

The final merged data set of single-cell myeloid or T/NK cell transcriptomes was analyzed using the SCANPY pipeline (64), including the LogNormalize method for normalization and the vst method for selection of highly variable features, scaling, and principal component analysis. The Harmony algorithm (65) was used for integration of data sets across the 3 distinct participants. Using the Harmony-corrected principal components (PCs), a 2-dimensional embedding was created using the Python implementation of UMAP (66). These embeddings appear in Figures 6 and 7. Clustering of the cells was performed as a continuation of the Seurat pipeline using the FindNeighbors function with the first 30 PCs as input and FindClusters function. Marker genes for each cluster with logFC and *P* values were defined using the FindMarkers function in Seurat. Marker genes selected for display on the heatmap in Figures 6 and 7 were from representative pathways among the genes showing adjusted *P* value < 0.001.

### Data availability.

NanoString data generated for this manuscript have been deposited in NCBI’s Gene Expression Omnibus (accession GSE200988).

### Statistics.

Statistical analysis for comparison of immune and epithelial cell density and for simple and multivariable linear regression was performed using Prism software version 9.0 (GraphPad). Any previously published immune cell quantifications were excluded from analysis. Specifically, the previously published quantifications of neutrophils and CD4^+^, CD8^+^, and CD19^+^ lymphocytes (22) were excluded for 5 out of the 24 COVID-19 patients and 3 out of the 12 controls (Figure 5B). Quantifications of these patients’ epithelial cells (Figure 3, B and C), CD4^+^ macrophages, CD4^–^ macrophages, and GZMB expression (Figure 5, B and C) have not been previously published and thus were not excluded from analysis. Calculation of *P* values was performed as indicated in the figure legends with 2-sided hypothesis testing. A *P* value less than 0.05 was considered significant. Error bars show median ± interquartile range. For the multivariable analysis the *P* values for each variable were calculated using the *t* statistic with 2-sided hypothesis testing. For the gene expression profiling of lung tissue across COVID-19 mortality cases and controls, *P* values were calculated using the Rosalind platform for nCounter data analysis and adjusted for multiple comparisons using the Benjamini-Hochberg method of estimating FDRs.

### Study approval.

Use of organ donor tissues and autopsy material does not qualify as “human subjects” research, as confirmed by the Columbia University IRB, as tissue samples were obtained from brain-dead and deceased individuals. All patient samples in this study were enrolled on protocols approved by the IRB at CUIMC.

## Author contributions

MC coordinated sample acquisition, processed samples, collected and analyzed the data, and created figures. SS contributed to writing and editing the paper, performed data analysis, and created figures. PA Szabo, SBW, JIG, and PD processed samples and analyzed data. PA Szabo and SBW processed samples for scRNA-Seq profiling and encapsulation using 10x Genomics Chromium. MRB monitored and consented ICU patients, oversaw clinical data analysis, and collected samples. TJC obtained and maintained IRB protocols, consented patients, and processed samples. MMLP and RM obtained and processed patient samples. MK, MES, and RM obtained and processed organ donor samples. AS and MMY provided lung autopsy samples and associated data and contributed to writing and editing the paper. AWK analyzed multiplex imaging of autopsies. PA Sims planned scRNA-Seq experiments and analyzed data. DLF oversaw compliance, coordinated sample acquisition, and helped edit the paper. SPW oversaw compliance, coordinated sample acquisition, planned experiments and analysis, and wrote and edited the paper.

## Supplementary Material

Supplemental data

Supplemental table 2

## Version 1 04/21/2022

In-Press Preview

## Version 2 06/08/2022

Electronic publication
