# Supplementary material for: Immune and epithelial determinants of age-related risk and alveolar injury in fatal COVID-19
Source: JCI Insight. 2022 Jun 8;7(11):e157608. doi: 10.1172/jci.insight.157608 (PMC9228710; doi:10.1172/jci.insight.157608)
Supplement: Supplemental data [file jciinsight-7-157608-s008.pdf]

Supplemental Figure 1

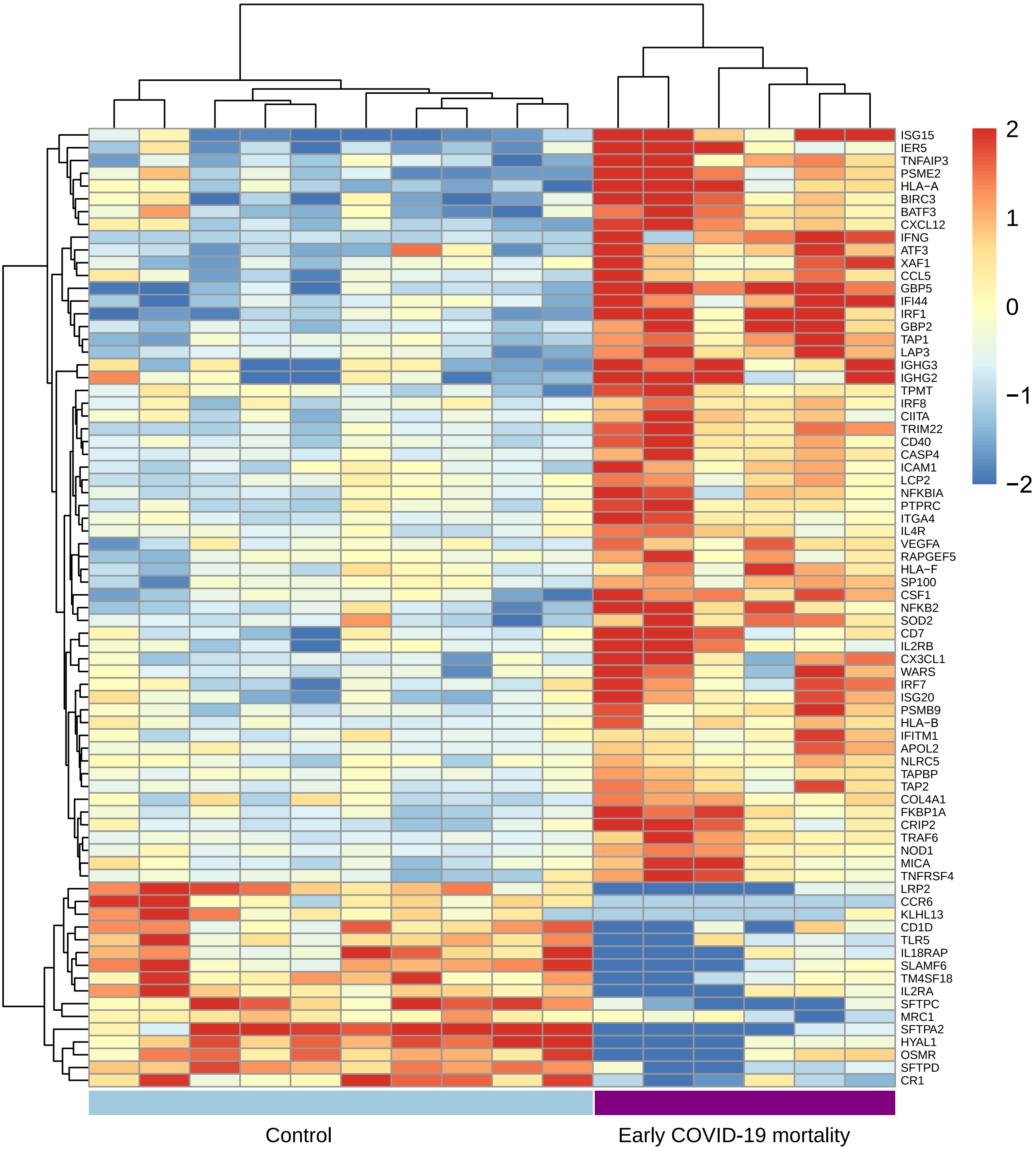

Supplemental Figure 1. Gene expression changes seen in early COVID-19 mortality cases compared to uninfected controls. Heatmap of normalized and scaled gene expression for all transcripts showing gene expression changes with absolute log fold change > 0.5 and adjusted P value < 0.05 in the comparison of early COVID-19 mortality cases (n = 6) and uninfected controls (n = 10). P values were calculated using the Rosalind platform for nCounter data analysis and adjusted for multiple comparisons using the Benjamini-Hochberg method of estimating false discovery rates (FDR).

Supplemental Figure 2

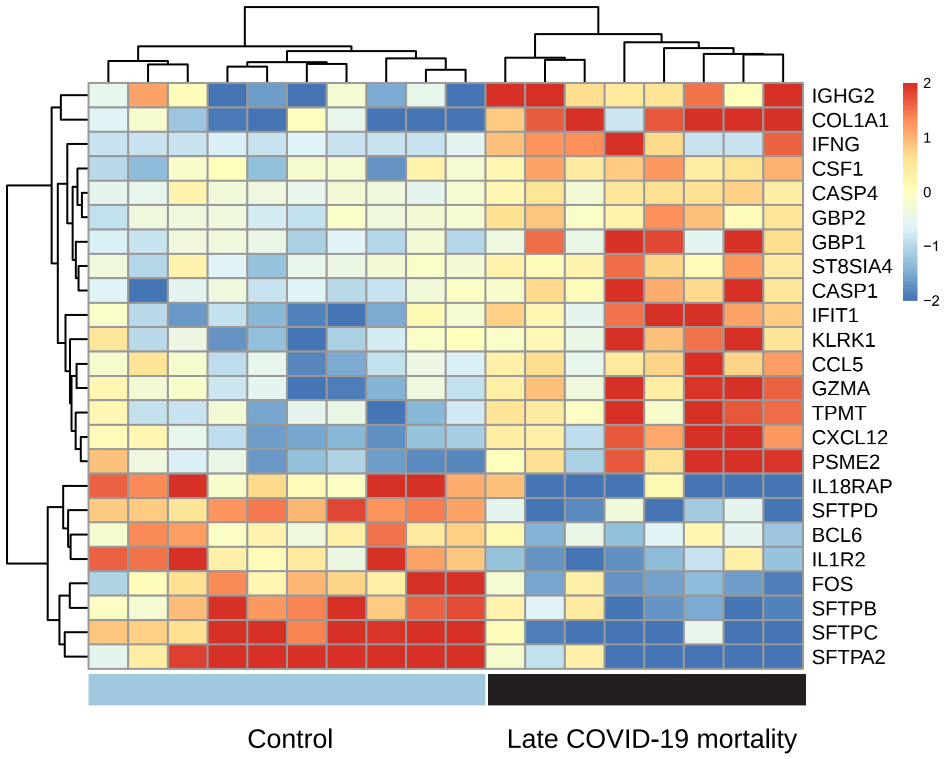

Supplemental Figure 2. Gene expression changes seen in late COVID-19 mortality cases compared to uninfected controls. Heatmap of normalized and scaled gene expression for all transcripts showing gene expression changes with absolute log fold change > 0.5 and adjusted P value < 0.05 in the comparison of late COVID-19 mortality cases (n = 8) and uninfected controls (n = 10). P values were calculated using the Rosalind platform for nCounter data analysis and adjusted for multiple comparisons using the Benjamini-Hochberg method of estimating false discovery rates (FDR).

Supplemental Figure 3

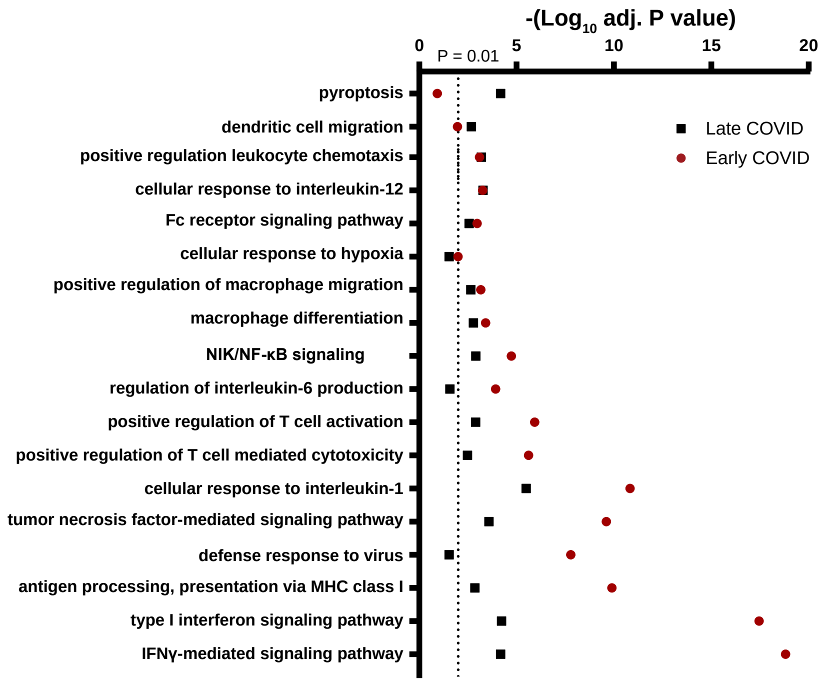

Supplemental Figure 3. Gene ontology analysis of transcripts altered in early and late COVID-19 mortality cases compared to controls. Gene ontology (GO) analysis showing the –(log10 adjusted P value) of representative pathways that are enriched for upregulated genes in the early COVID-19/control (red dots) and the late COVID-19/control (black squares) comparisons. The dotted line shows adjusted P value = 0.01. Adjusted P values were computed using the hypergeometric test.

Supplemental Figure 4

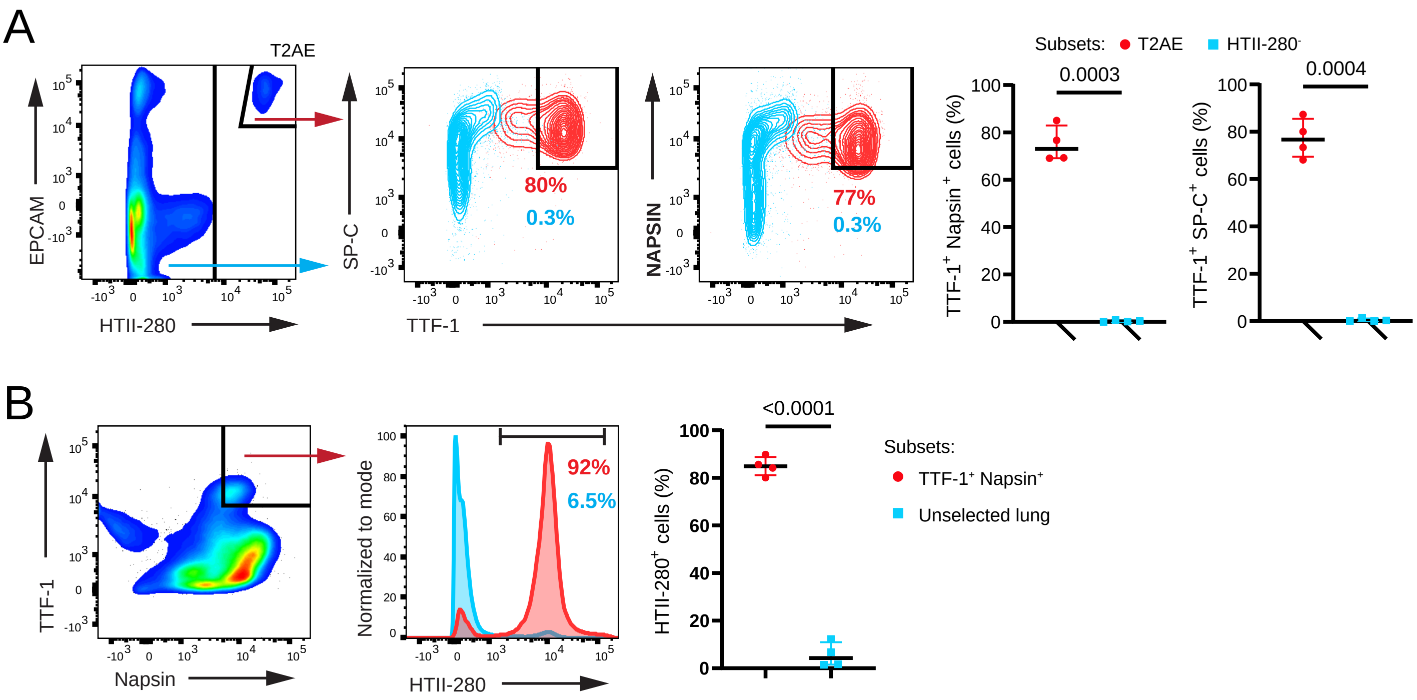

Supplemental Figure 4. Validation of TTF-1 and Napsin-A profiling for classification of pulmonary epithelial cells. (A) Flow cytometry profiles showing the gating strategy for identifying type 2 alveolar epithelial cells (T2AE) in a representative lung tissue single cell suspension. The plot on the left shows the epithelial cellular adhesion molecule (EPCAM) marker against the T2AE marker HTII-280. Sub-gating on the T2AE population (EPCAM+ HTII-280+) is shown in the contour plots to the right. The T2AE are shown in red and the other cells are shown in blue with the TTF-1 marker plotted against surfactant protein C (SP-C, left contour plot) and Napsin-A (right contour plot). The dot plots on the right show the compiled percentages of TTF-1+ SP-C+ cells (left dot plot) and TTF-1+ Napsin-A+ cells (right dot plot) within the T2AE gate (EPCAM+ HTII-280+) from 4 uninfected organ donors. (B) The plot on the left shows the TTF-1 marker against the Napsin-A marker in a representative lung tissue single cell suspension. Sub-gated TTF-1+ Napsin-A+ T2AE cells are shown in the histogram of HTII-280 expression to the right in red with all other cells in blue. The compiled percentages of HTII-280+ cells within the TTF-1+ Napsin-A+ subgate (red) compared to the other cells (blue) are shown in the dot plot on the right. The error bars show the median with interquartile range. P values were calculated using a two-tailed T-test.

Supplemental Figure 5

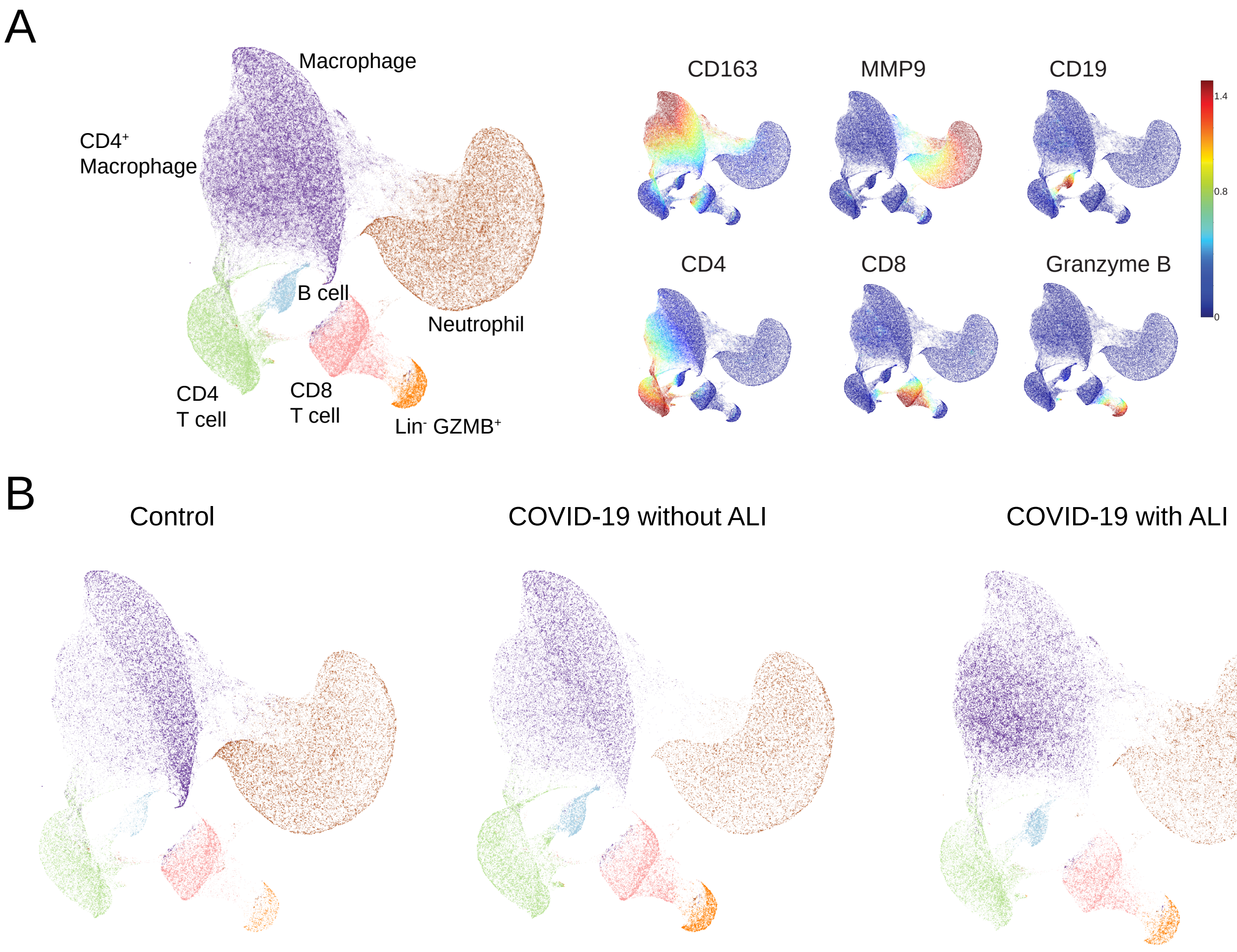

Supplemental Figure 5. Immune cell classification in lung tissue sections from COVID-19 mortality cases and controls. (A) UMAP embedding of all immune cells based on expression of the 6 immune cell lineage markers for all of the COVID-19 cases (n = 24) and controls (n = 12) is shown on the left with colors corresponding with the cell phenotypes that were separately determined for immune cell quantification using the inForm software and training a machine learning classifier. The normalized and scaled expression of each marker is shown superimposed on the UMAP to the right. (B) The UMAP embeddings, down sampled for equal representation of each subject, are shown of lung immune cells from the uninfected controls (n = 12, left), and the COVID-19 cases with histopathological diagnosis of acute lung injury (ALI) (n = 15, right), and no ALI (n = 9, right).

Supplemental Figure 6

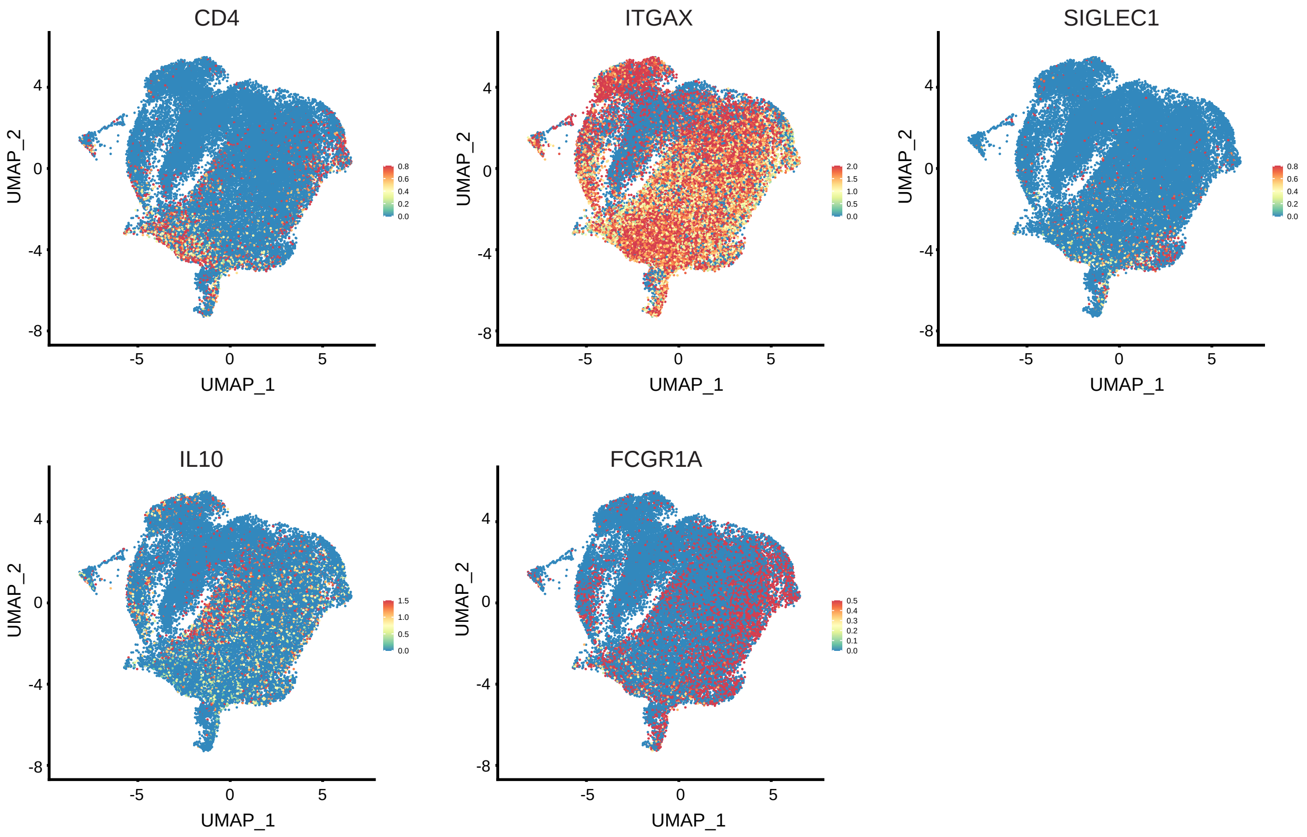

Supplemental Figure 6. Macrophage lineage marker expression in the myeloid cells from airway washes of intubated COVID-19 patients. (A) UMAP embeddings of total myeloid cells from airway of three intubated COVID-19 patients with feature plots showing normalized expression of *CD4* (top, left), *ITGAX* (encoding CD11c) (top, middle), *SIGLEC1* (encoding CD169) (top, right), *IL10* (bottom, left) and *FCGR1A* (encoding CD64), (bottom, right).

Supplemental Table 1.

	**Early (n=8)**	**Late (n=16)**
**Clinical Characteristics**		
Age, years median (range)	71.5 (59-93)	75.5 (57-90)
Sex, male, (%)	7 (87.5%)	11 (68.8%)
Weight (kg), median (IQR)	91.7 (72.9-99.7)	76.7 (67.9-93.3)
Body Mass Index, kg/m2, median (IQR)	30.0 (26.1-35.1)	27.6 (23.9-33.2)
Intubation, (%)	6 (75%)	8 (50%)
Hospitalization, Days, median (range)	0.5 (0-5)	16 (0 -33)
Total time from symptoms to death, Days, median (range)	2.5 (1 -9)	19.5 (10- 43)
**Pathological Characteristics**		
Acute Lung Injury	2 (25%)	13 (81.3%)
Capillary Proliferation	6 (75%)	7 (43.8%)
N-protein	4 (50%)	4 (25%)
**Race or Ethnic Group, (%)**		
Hispanic	2 (25%)	9 (56.3%)
African American	1 (12.5%)	1 (6.3%)
White	0 (0)	1 (6.3%)
Other or Unknown	5 (62.5%)	5 (31.3%)
**Co-Morbidities, (%)**		
Hypertension	8 (100%)	15 (93.8%)
Diabetes	3 (37.5%)	9 (56.3%)
Preexisting Heart Disease	4 (50%)	7 (43.8%)
Congestive Heart Failure	2 (25%)	3 (18.8%)
Coronary Artery Disease	2 (28.6%)	6 (37.5%)
Preexisting Lung Disease	2 (25%)	3 (18.8%)
Hyperlipidemia	2 (25%)	6 (37.5%)
**Laboratory Values, Median (range)**		
Oxygen saturation % at presentation	77 (30%-91%)	83 (66%-99%)
Lowest PaO_2 (mm Hg)	62 (37-87)	53 (23-79)

Abbreviations: PaO2, arterial partial pressure of oxygen; IQR, interquartile range; N-protein, nucleocapsid protein

Supplemental Table 3.

Variable	T2AE			T1AE		
	Regression coefficient	SE	P value	Regression coefficient	SE	P value
Donor age	-0.01272	0.004134	0.0039	-0.00236	0.004684	0.6173
Smoking history	0.2569	0.1612	0.1193	-0.009607	0.1826	0.9583
Underlying lung disease	-0.7302	0.279	0.0127	-0.379	0.3162	0.238

Abbreviations: SE, standard error; T2AE, type 2 alveolar epithelial; T1AE, type 1 alveolar epithelial

Supplemental Table 4.

Variable	Alveolar epithelial cell density $\ln(\text{TTF-1}^+ \text{cells/mm}^2)$		
	Regression coefficient	SE	P value
$GZMB^+$ (% of CD8)	-0.01929	0.007198	0.0148
Patient age	0.007893	0.01285	0.5465
ALI	-0.9576	0.2982	0.0046
Symptomatic interval (days)	-0.03287	0.01228	0.0149

Abbreviations: SE, standard error; GZMB, granzyme B; ALI, acute lung injury

Supplemental Table 5.

Variable	Alveolar epithelial cell density ln(TTF-1$^+$ cells/mm^2)		
	Regression coefficient	SE	P value
CD4$^+$ macrophage (cells/mm^2)	-0.0004747	0.000129	0.0016
Patient age	0.01671	0.01065	0.1332
ALI	-0.958	0.257	0.0014
Symptomatic interval (days)	-0.009828	0.01094	0.3802

Abbreviations: SE, standard error; ALI, acute lung injury

Supplemental Table 6.

Variable	Alveolar epithelial cell density ln(TTF-1^{+} cells/mm^{2})		
	Regression coefficient	SE	P value
Macrophage CD4 expression	-1.715	0.6556	0.017
Patient age	0.01918	0.0119	0.1235
ALI	-1.12	0.2771	0.0007
Symptomatic interval (days)	-0.01044	0.01252	0.4147

Abbreviations: SE, standard error; ALI, acute lung injury

Supplemental Table 7.

Marker	Application	Clone	Supplier	Cat#
Human TTF-1	IHC	8G7G3/1	Cell Marque	343M-97
Human Napsin A	IHC	Rabbit polyclonal	Biocare Medical	PP434AA
ChromoPlex 1 Dual Detection for BOND	IHC	n/a	Leica	DS9665
SARS-CoV/SARS-CoV-2 Nucleocapsid Antibody	IHC	001	SinoBiological	40143-R001
FITC anti-Human IgG	Immunofluorescence	Rabbit polyclonal	Dako	F 0202
Human Napsin A	Flow cytometry	Rabbit polyclonal	ThermoFisher-Invitrogen	PA5-114325
Human Surfactant Protein C	Flow cytometry	Rabbit polyclonal	ThermoFisher-Invitrogen	BS-10067R
APC anti-rabbit IgG F(ab')$_2$ Fragment	Flow cytometry	Donkey polyclonal	JacksonImmuno	711-136-152
HTII-280	Flow cytometry	HTII-280	Terrace Biotech	TB-27AHT2-280
AF488 anti-mouse IgM F(ab')$_2$ Fragment	Flow cytometry	Goat polyclonal	JacksonImmuno	115-546-075
Brilliant Violet 605™ anti-human CD326 (EpCAM) Antibody	Flow cytometry	9C4	Biolegend	324224
Anti-human CD19	Multispectral staining (Opal 540)	BT51E	Leica	CD19-163-L-CE
Anti-human CD8	Multispectral staining (Opal 690)	4B11	Leica	CD8-4B11-L-CE
Anti-human CD163	Multispectral staining (Opal 650)	10D6	Leica	CD163-L-CE
Anti-human CD4	Multispectral staining (Opal 520)	EPR6855	Abcam	ab133616
Anti-human GzmB	Multispectral staining (Opal 570)	11F1	Leica	GRAN-B-L-CE
Anti-human MMP9	Multispectral staining (Opal 620)	L51/82	Biolegend	819701
Opal 7-Color Automated IHC Detection Kit	Multispectral staining	n/a	n/a	NEL821001KT
